# Mechanisms for leaf color changes in *Osmanthus fragrans* ‘Ziyan Gongzhu’ using physiology, transcriptomics and metabolomics

**DOI:** 10.1186/s12870-023-04457-8

**Published:** 2023-09-27

**Authors:** Peng Guo, Ziqi Huang, Wei Zhao, Nan Lin, Yihan Wang, Fude Shang

**Affiliations:** 1 College of Life Science, Henan Agricultural University, Zhengzhou, 450046 Henan China; 2 Henan Engineering Research Center for Osmanthus Germplasm Innovation and Resource Utilization, Zhengzhou, 450046 Henan China

**Keywords:** *Osmanthus fragrans* ‘Ziyan Gongzhu’, RNA-seq, Metabolomics, Chlorophyll, Carotenoid, Anthocyanin, *OfMYB* genes

## Abstract

**Background:**

Color-leaved *O. fragrans* is a variety of *Osmanthus fragrans*, which has both the fragrance of *Osmanthus* and the color of color-leaved plants. However, the molecular mechanism of color change of color-leaved *O. fragrans* is not clear. In this study, we analyzed the regulatory mechanism of four different color leaves of ‘Ziyan Gongzhu’ through physiological, transcriptome and metabolome levels.

**Results:**

Firstly, we measured the leaf pigments content and leaf chromatic parameters for correlation analysis, indicating a significant correlation between them. Overall, the content of chlorophyll a + b is low and the content of anthocyanin is high in T1 and T2 leaves, along with low expression of chlorophyll synthesis genes (HEMA, CHLG, and CAO, etc.) and high expression of anthocyanin synthesis genes (F3H, F3’H, DFR and ANS, etc.), resulting purple red and light purple in T1 and T2 leaves, respectively. It was also found that the pigment closely related to the color leaves of ‘Ziyan Gongzhu’ was cyanidin. The content anthocyanins, may be regulated by two putative MYB activators (*OfMYB3* and *OfMYB4*) and two putative MYB repressors (*OfMYB1* and *OfMYB2*). In contrast, the content of chlorophyll a + b is high and the content of anthocyanin is low in T3 and T4 leaves, along with high expression of chlorophyll synthesis genes and low expression of anthocyanin synthesis genes, resulting yellow green and dark green in T3 and T4 leaves, respectively. And abnormal chloroplast development affects chlorophyll content in T1, T2, and T3 leaves. Although the content of carotenoids first dropped in T2 leaves, it then rapidly accumulated in T4 leaves, in sync with the increase in the expression of genes related to carotenoid biosynthesis (ZDS, LHYB, and ZEP, for example). Analysis of photosynthetic, carbohydrate and hormone-related differentially abundant metabolites (DAMs) and DEGs found that they may participate in the regulation of leaf color change of ‘Ziyan Gongzhu’ by affecting pigment synthesis.

**Conclusion:**

Our results pave the way for a comprehensive knowledge of the regulatory processes governing leaf color in ‘Ziyan Gongzhu’ and identify possible genes for application regarding molecular colored-leaf cultivar breeding.

**Supplementary Information:**

The online version contains supplementary material available at 10.1186/s12870-023-04457-8.

## Introduction

*Osmanthus fragrans* is a plant of *Osmanthus* in Oleaceae, one of the top 10 traditional Chinese flowering plants, with high ornamental value [1]. In 2014, the International Registration Center of *Osmanthus* Species established a new variety group of color-leaved *Osmanthus* on the basis of the original four varieties groups, namely, Asiaticus, Albus, Luteus, and Aurantiacus [2]. In recent years, 53 varieties of color-leaved *Osmanthus* have successively passed the examination and approval. For example, the new cultivar ‘Ziyan Gongzhu’ produces purple red (RHS 67A) buds, stems, and leaves. The leaves gradually lose their purple red color, turn light purple (RHS 65A) to yellow green (RHS 1C), and become dark green (RHS N137A) as they develop. The colorful leaves last for almost half a year. Hence, these cultivars with hued leaves extensively provide germplasm for the development of new ones. Yet, the fundamental processes of leaf color change remain unclear.

Including carotenoid production, chlorophyll, photosynthesis, secondary metabolite synthesis, and chloroplast growth, changing leaf color is complicated [3]. In general, changes in the synthesis, degradation, content and proportion of chlorophyll, anthocyanins and carotenoids directly lead to abnormal leaf color. Anthocyanins represent as the last result of biosynthesizing flavonoid, depicting crucial functions in purple or red pigmentation of plant leaves [4–6]. For example, previous studies have found that the content of anthocyanins in the purple leaves of *Camellia sinensis* is mainly affected by *CsUGT* [7]*.* For plants, anthocyanin synthesis is robustly affected by the metabolic pathway’s structural genes, which directly encode the enzymes necessary in the pathway, such as phenylalanine ammonia-lyase (PAL), chalcone synthase (CHS), flavanone 3-hydroxylase (F3H), dihydroflavonol 4-reductase (DFR), and anthocyanin synthase (ANS) [8, 9]. Transcription factors regulate the spatiotemporal expression and expression intensity of genes in metabolic pathways by combining with cis-acting elements of structural gene promoter to regulate the coloration of plant leaves. MYB (v-myb avian myeloblastosis viral oncogene homolog), bHLH (basic helIX-loop-helIX) and WDR (WD40 repeat proteins) play a major role in the regulation of anthocyanin synthesis pathway at the transcription level, which usually directly regulate the transcription of structural genes in the form of MBW complexes [10].

Many studies have shown that chlorophyll is the most influential factor in determining leaf color phenotype. Chlorophyll biosynthesis pathway of higher plants includes 15 steps of enzyme catalyzed reaction. No matter which node is blocked in the process, it may cause changes in the content and proportion of chlorophyll, thus causing changes in leaf color [11]. For plants, chlorophyll synthesis is robustly affected by the metabolic pathway’s genes, which directly encode the enzymes necessary in the pathway, such as Glutamyl-tRNA reductase (GluTR), Glutamyl-tRNA reductase (HEMA) and Magnesium chelatase (MgCh), etc. Coproporphyrinogen III oxidase (CPOX) and protoporphyrinogen oxidase (PPOX) are also key enzymes in the process of plant chlorophyll biosynthesis. The production of many plant chlorophyll deletion mutants has been proved to be related to these two enzymes [12]. In addition, studies on *Arabidopsis*, *Oryza sativa* and *Solanum lycopersicum* mutants showed that chloroplast development also affected the phenotype of chlorotic leaves.

Carotenoids are ubiquitous in plants. They are generally expressed in the form of red, orange red and yellow, and play a light protective role in photosynthesis. The carotenoid content has a certain influence on the color of leaves. Phytoene synthase (PSY) is a key regulator of carotenoid accumulation in plants [13]. For plants, carotenoids synthesis is robustly affected by the metabolic pathway’s genes, which directly encode the enzymes necessary in the pathway, such as phytoene desaturase (PDS), ζ-carotene isomerase (Z-ISO), ζ-carotene desaturase (ZDS) and carotene isomerase (CRTISO).

Here, we study the new colored-leaf cultivar ‘Ziyan Gongzhu’ color leaf regulation mechanism from the level of physiological characteristics, transcriptome and metabolome, excavates key regulatory genes, comprehensively analyzes the color regulation mechanism of color-leaved *Osmanthus*, clarifies the key gene regulation network and metabolic pathway.

## Materials and methods

### Plant materials

**Fig. 1:**
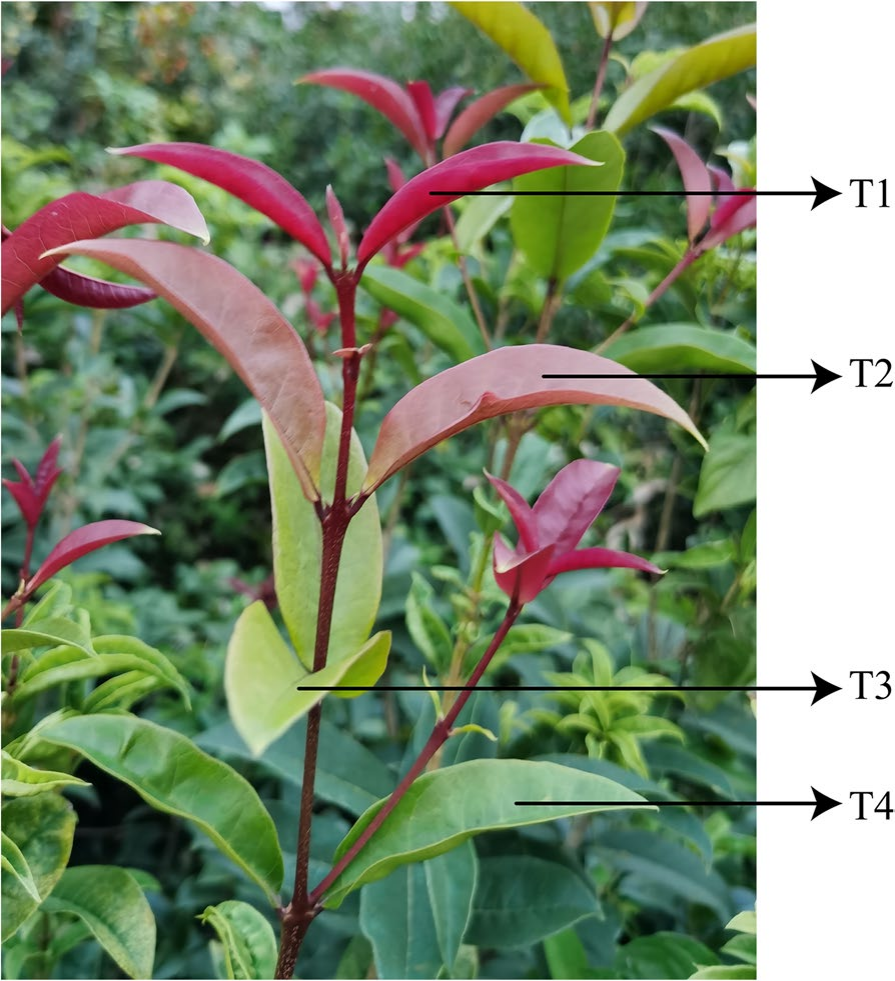
Leaf characteristics of ‘Ziyan Gongzhu’ at the four developmental stages. (T1: purple red leaf (RHS 67A), T: stage, T2: light purple leaf (RHS 65A), T3: yellow green leaf (RHS 1C), T4: dark green leaf (RHS N137A)

The experimental material is two-year ‘Ziyan Gongzhu’ cuttings, taken from the *Osmanthus fragrans* germplasm resource bank (Zhejiang, China). The leaf color of ‘Ziyan Gongzhu’ changes from young leaves to mature leaves are purple red (T1, T: stage, RHS 67A), light purple (T2, RHS 65A), yellow green (T3, RHS 1C), dark green (T4, RHS N137A) (Fig. 1). The cutting seedlings of ‘Ziyan Gongzhu’ with good growth status were measured with chlorophyll fluorescence parameters (3 biological replications × 4 stages) before collecting leaves for subsequent experiments. The leaves of T1, T2, T3 and T4 were collected at noon on a sunny day and frozen in liquid nitrogen, then stored at -80 ℃ for metabolome (6 biological replications × 4 stages) and transcriptome sequencing (3 biological replications × 4 stages), and fresh leaves were used for measurement of some physiological indicators (3 biological replications × 4 stages).

### Determination of pigment content

The content of chlorophyll and carotenoid is determined according to Peng (1992) [14] and Li (2000) [15]. Three biological replicates were performed for each developmental stage. The calculation formula is as follows:

$$\text{Chlorophyll a content (mg/g)}\ {:} Chl\ a = 0.1^{\ast}(9.78OD_{\mathit{663}} -0.99OD_{\mathit{645}})$$

$$\text{Chlorophyll b content (mg/g)}\ {:}\ Chl\ b = 0.1^{*}(21.43OD_{\mathit{645}} - 4.65OD_{\mathit{663}})$$

$$\text{Chlorophyll a+b content (mg/g)}\ {:}\ Chl\ a\!+\!b = 0.1^{*}(5.13OD_{\mathit{663}} +20.44OD_{\mathit{645}})$$

$$\text{Carotenoid content (mg/g)}\ {:}\ Car = 0.1^{*}(4.7OD_{\mathit{440}} -0.27 Chl\ a\text{+}b)$$

The content of anthocyanins is determined according to Pietrini and Massacci (1998) [16] and Li (2000) [15]. Three biological replicates were performed for each developmental stage. The calculation formula is as follows (FW: Fresh weight):

$$\text{Relative content of anthocyanins (mg/g)} =OD_{\mathit{535}}/0.1^{\ast} FW$$

### Leaf color measurement

The colors of fresh leaves were measured with a NF555 chroma meter (Nippon Denshoku, Tokyo, Japan). Three parameters, L*, a*, and b* were determined: L* (ranging from 0 to 100) indicates lightness. Positive and negative values of a* standard for red and green, respectively, and those of b* represent yellow and blue, respectively [17]. Five biological replicates were performed for each developmental stage. Correlation analysis of leaf color parameters and pigment content results using Pearson.

### Determination of pH

The pH of leaves is determined according to Tang (2006) [18]. Three biological replicates were performed for each developmental stage.

### Observation of chloroplast ultrastructure

To conduct transmission electron microscopy (TEM), fresh leaves were sectioned (1 mm^3^) along the central vein and fixed for 2 h at room temperature in Servicebio fixative (Wuhan, China). And then wash the tissues using 0.1 M Phosphate buffer (pH 7.4) for 3 times, 15 min each. Tissues avoid light post fixed with 1% OsO4 in 0.1 M PB (pH 7.4) for 7 h at room temperature. After remove OsO4, the tissues are rinsed in 0.1 M PB (pH 7.4) for 3 times, 15 min each. Then dehydration was carried out with acetone of different concentrations of 30%, 50%, 70%, 80%, 95%, 100% and 100% from low to high for 1 h each time. Absolute ethanol and acetone elute for 0.5 h each time according to the volume ratio of 3:1, 1:1 and 1:3, and then elute with acetone for 1 h. Acetone and EMBed 812 (SPI, America) infiltrate for 2–4 h, overnight and 2–4 h according to the gradient of 3:1, 1:1 and 1:3 by volume, respectively. 5–8 h at 37 °C with pure 812 embedding agent. Pour the pure EMBed 812 into the embedding models and overnight at 37 °C, implant the tissues into the pure EMBed 812. The embedding models with resin and samples were polymerized in an oven at 65 °C for over 48 h. Before removal and cooling to room temperature, the resin was polymerized. With a UC7 ultramicrotome from Leica Microsystems GmbH (Wentzler, Germany), 60–80 nm thick pieces were sliced. To prevent CO2, the slices were stained for eight minutes alongside lead citrate and 2% uranium acetate saturated alcohol solution. A Hitachi HT7700/HT7800 TEM (Tokyo, Japan) studied and performed ultrastructure imaging. Average number of chloroplasts per cell was determined by determining the number of chloroplasts in five randomly selected cells. Chloroplast length and breadth among five intact ones from various cells were assessed.

### Measurement of chlorophyll fluorescence parameters

The MINI-PAM-II (WALZ, Germany), an ultraportable modulated chlorophyll fluorescence meter, was used to measure the fluorescence parameters. The initial fluorescence F0 and maximum fluorescence Fm, which reflected various fluorescence yields when the PSII reaction center was completely opened or closed, were measured 15 min after different color leaves of plants had acclimated to the dark. All chlorophyll fluorescence parameters were analyzed with WinControl-3 software (WALZ, Germany).

### RNA extraction, RNA-Seq and RNA-Seq analysis

Total RNA was extracted from ~ 200 mg of frozen leaves with different colors in four stages using Trizol reagent kit (Invitrogen, Carlsbad, CA, USA). Each stage had 3 biological replicates. RNA-Seq was made at Guangzhou Gene Denovo Biotechnology Co. (Guangzhou, China) using an Illumina HiSeq platform (Illumina Inc., San Diego, CA, USA). High-quality clean reads were then mapped to an *O. fragrans* reference genome [19] using HISAT v2.1.0 software. Screening criteria for differentially expressed genes (DEGs) were | log2(fold change) |≥ 1 and FDR < 0.05. Gene functions were annotated according to Kyoto Encyclopedia of Genes and Genomes (KEGG) [20], National Centre for Biotechnology Information (NCBI) non-redundant protein sequences (NR), Gene Ontology (GO).

### Identification and phylogenetic analyses of *OfMYBs*

The putative *OfMYB* genes associated with flavonoids biosynthesis in ‘Ziyan Gongzhu’ leaves were identified after initially screening of the differentially expressed *OfMYBs*. To categorize and survey the evolutionary relationships of *OfMYBs*, multiple sequences of the selected *OfMYBs* and 26 MYBs from other plant species were aligned using ClustalW, and a phylogenetic tree was then constructed using MEGA 7.0, with the maximum-likelihood method [21]. GenBank accession numbers of the MYBs used in the phylogenetic analyses are shown in Table S1.

### RT-PCR confirmation

Thirty DEGs were randomly selected for RT-PCR confirmation. The sequences of the Forward and Reverse primers were listed in Table S2. Actin was selected as an internal standard. RT-PCR was performed in a reaction mixture with a total volume of 20 µL. Each reaction mixture consisted of 2 µL of cDNA template, 1 µL of each primer, 6 µL ddH_2_O and 10 µL SYBR Premix Ex Taq mix (Vazyme, Nanjing, China). Amplification reactions involved an initial denaturation step at 95 ℃ for 30 s, followed by 40 cycles of 10 s at 95 ℃, 30 s at 60 ℃. Using 2^−ΔΔCt^, gene relative expressions were determined relative to those from internal control. Every gene has a triad of biological replications.

### Nontargeted metabolome in leaves

Nontargeted metabolomics were performed at Gene Denovo Co., Ltd. (Guangzhou, China). 100 mg of each powdered leaves were ground with liquid nitrogen, and the homogenate was thoroughly vortexed with precooled 80% methanol and 0.1% formic acid. The samples were put on ice for five minutes before being centrifuged for twenty minutes at 15,000 g and 4 °C. Certain supernatants were diluted with LC–MS grade water to a final concentration of 53% methanol. The samples were then transferred to a fresh Eppendorf tube and centrifuged for 20 min at 4 °C and 15,000 g. Injecting the supernatant into the UPLC-MS/MS apparatus (UPLC, Vanquish UHPLC, Thermo Fisher, Germany; MS, Q Exactive™ HF-X, Thermo Fisher, Germany) analysis. Metabolite identification was performed using mzCloud (https://www.mzcloud.org/. Accessed 20 May 2020), mz Vault and MassList database. Differentially abundant was a metabolite with VIP ≥ 1 in OPLS-DA and *p* < 0.05. Using the KEGG database, the activities of differentially abundant metabolites (DAMs) were annotated.

### Conjoint analysis of metabolomics and transcriptomics

This work connected all differentially expressed genes and metabolites to the KEGG pathway database, enabling gene and metabolite integration analysis. And performed a Two-way Orthogonal PLS (O2PLS) analysis using the OmicsPLS package of R.

### Statistical analysis

SPSS Statistics 24 (IBM, Armonk, NY, USA) was used for data analysis. Every measurement was performed in triplicate. The findings were the mean ± standard error of three to six replicates. Using a one-way analysis of variance (ANOVA), the means of the measured parameters were compared. Least significant difference (LSD) post-hoc test was used to examine the statistical significance of the mean differences (*P* < 0.05). For principal component analysis (PCA), hierarchical Cluster Analysis (HCA), OPLS-DA and two-way Orthogonal Partial Least Square (O2PLS) analysis, the data were normalized and analyzed by R software (https://www.r-project.org/. Accessed 23 November 2021).

## Results

### Pigment concentrations in leaves

**Fig. 2:**
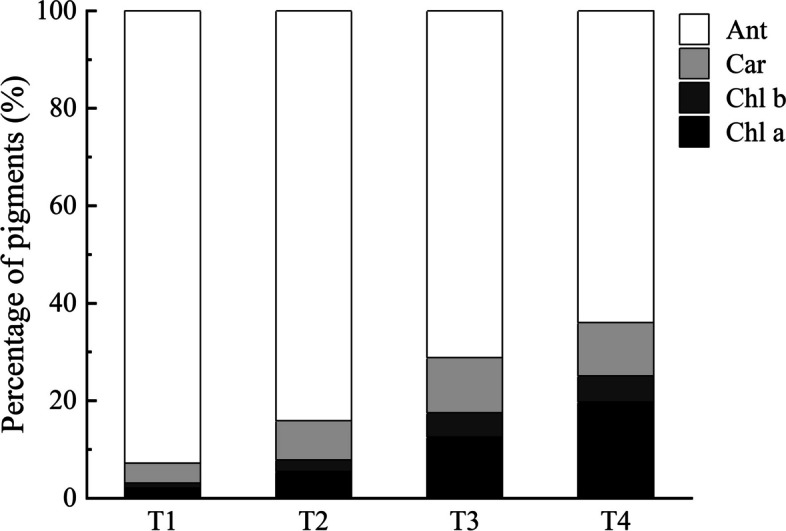
Percentage of pigments content in leaves of ‘Ziyan Gongzhu’ at the four developmental stages. Chl a, Chlorophyll a content; Chl b, Chlorophyll b content; Car, Carotenoid content; Ant, Anthocyanin content. (T1: purple red leaf (RHS 67A), T2: light purple leaf (RHS 65A), T3: yellow green leaf (RHS 1C), T4: dark green leaf (RHS N137A)

Pigment concentrations in leaves of ‘Ziyan Gongzhu’ were measured at the four different stages. Table 1 shows that the content of chl a + b is highest in T4 (1.99 mg/g) and lowest in T1 (0.24 mg/g), the content of chl a + b in T2 and T3 is 0.26 mg/g and 0.56 mg/g, respectively. The content of carotenoid is highest in T4 (0.87 mg/g) and similar levels were maintained among T1 (0.31 mg/g), T2 (0.27 mg/g) and T3 (0.36 mg/g). Meanwhile, the content of anthocyanin is highest in T1 (7.19 mg/g) and followed by T4 (5.08 mg/g), and similar levels were maintained between T2 (2.83 mg/g) and T3 (2.27 mg/g). The ratios of Car/Chl a + b and Ant/Chl a + b both decrease as the leaves turn green. Anthocyanidin content accounts for the largest percentage of pigments content in T1 leaves (Fig. 2). At this time, the leaves are purple red. As the colored leaves turn green, the percentage of anthocyanidin content in the leaves gradually decreases, and the percentage of chlorophyll content gradually increases, which is mainly determined by the content of chlorophyll a.

**Table 1:** Comparison of pigment content in leaves of ‘Ziyan Gongzhu’ at the four developmental stages

	Chl a + b^a^ (mg/g)	Car^b^ (mg/g)	Ant^c^ (mg/g)	Car/Chl a + b	Ant/Chl a + b
T1	0.24 ± 0.00c	0.31 ± 0.01c	7.19 ± 0.11a	1.31 ± 0.04a	29.90 ± 0.58a
T2	0.26 ± 0.01c	0.27 ± 0.00d	2.83 ± 0.13c	1.02 ± 0.02b	10.74 ± 0.71b
T3	0.56 ± 0.22b	0.36 ± 0.00b	2.27 ± 0.04d	0.65 ± 0.02c	4.06 ± 0.11c
T4	1.99 ± 0.02a	0.87 ± 0.01a	5.08 ± 0.07b	0.44 ± 0.00d	2.55 ± 0.05c

Chl a + b^a^, Chlorophyll a + b content; Car^b^, Carotenoid content; Ant^c^, Anthocyanin content. Different lowercase letters indicate a significant difference (*p* < 0.05) relative to the value at the T4, as determined using ANOVA analysis, which is based on Duncan’s multiple range test. (T1: purple red leaf (RHS 67A), T2: light purple leaf (RHS 65A), T3: yellow green leaf (RHS 1C), T4: dark green leaf (RHS N137A)

### Leaf chromatic parameters

**Fig. 3:**
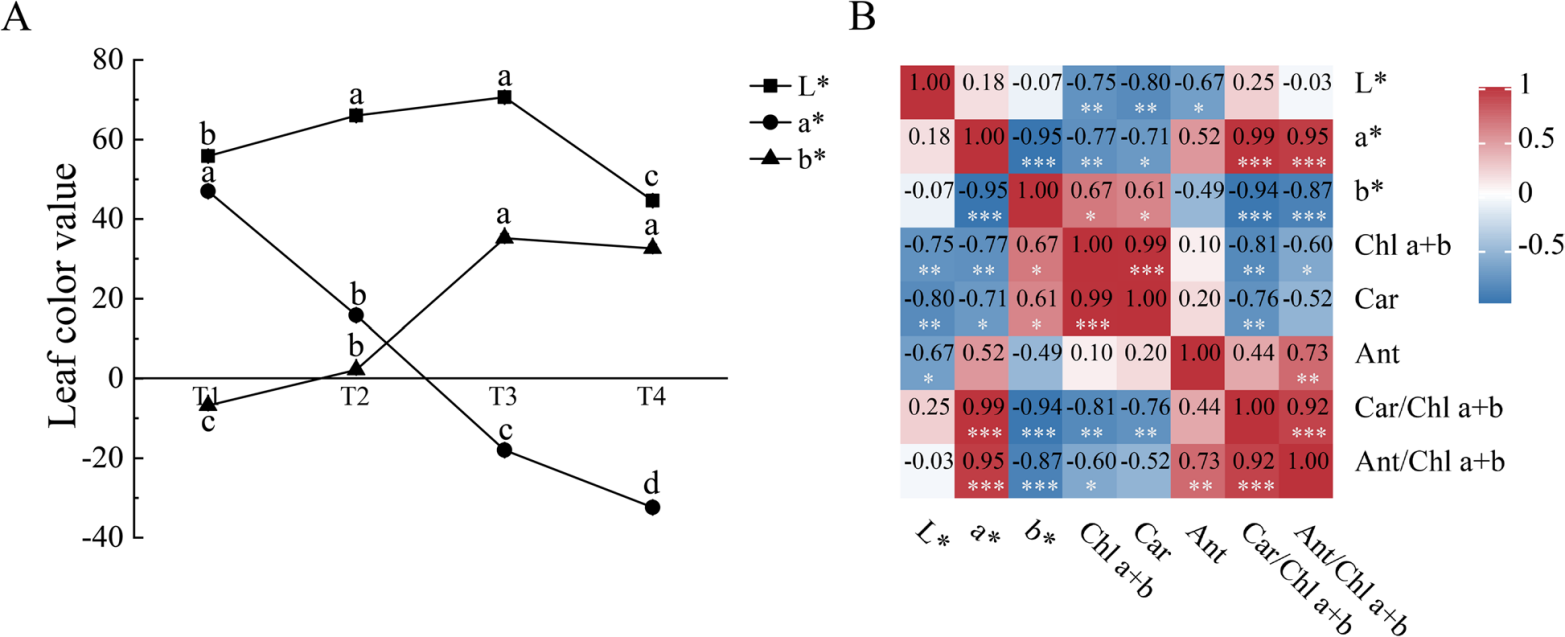
CIE Lab value (L, a*, b*) (**A**) and correlation analysis results between leaf chromatic parameters and pigment content and ratio (**B**) of ‘Ziyan Gongzhu’ leaves at the four developmental stages. Different lowercase letters indicate a significant difference (*p* < 0.05) relative to the value at the T4, as determined using ANOVA analysis, which is based on Duncan’s multiple range test. Chl a + b, Chlorophyll a + b content; Car, Carotenoid content; Ant, Anthocyanin content. *, *p* < 0.05; **, *p* < 0.01; ***, *p* < 0.001. (T1: purple red leaf (RHS 67A), T2: light purple leaf (RHS 65A), T3: yellow green leaf (RHS 1C), T4: dark green leaf (RHS N137A)

The measurement results of CIE Lab value (L*, a*, b*) of ‘Ziyan Gongzhu’ leaves at the four developmental stages are shown in Fig. 3A. T1 leaves are purple red, with the highest value of a* and the lowest value of b*. T3 leaves are yellow green, with L* and b* being the highest, while T4 leaves are dark green, with L* and a* having the lowest values. The correlation analysis results between leaf chromatic parameters and pigment content and ratio are shown in the Fig. 3B. The contents of chlorophyll a + b, carotenoid and anthocyanidin are extremely significantly and significantly negatively correlated with the value of L*, respectively. The ratios of Car/Chl a + b and Ant/Chl a + b is extremely significantly positively correlated with the value of a*; and the contents of chlorophyll and carotenoid are extremely significantly and significantly negatively correlated with the value of a*, respectively. The ratios of Car/Chl a + b and Ant/Chl a + b is extremely significantly negatively correlated with the value of b*; and the contents of chlorophyll and carotenoid are significantly positively correlated with the value of b*, respectively.

### pH of leaves

**Fig. 4:**
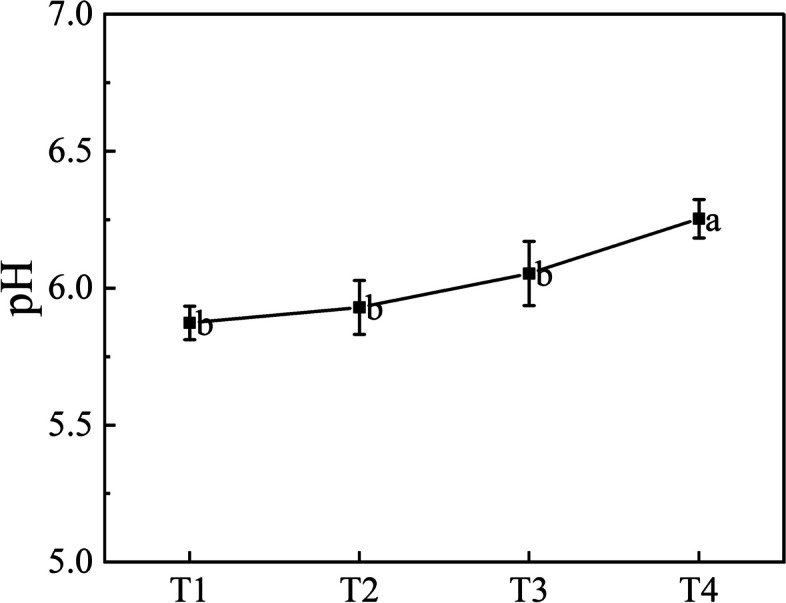
The pH of ‘Ziyan Gongzhu’ leaves at the four developmental stages. Different lowercase letters indicate a significant difference (*p* < 0.05) relative to the value at the T4, as determined using ANOVA analysis, which is based on Duncan’s multiple range test. (T1: purple red leaf (RHS 67A), T2: light purple leaf (RHS 65A), T3: yellow green leaf (RHS 1C), T4: dark green leaf (RHS N137A)

The color expression of anthocyanidin is affected by the pH of plant cell fluid, which is red under acidic conditions and blue under alkaline conditions [22]. The pH of the leaves of 'Ziyan Gongzhu' increased slowly with the leaves turning green (Fig. 4). The pH value increased from 5.87 to 6.25 in the process of leaf color changing from T1 purple red to T4 dark green, but all in the acidic range.

### Observation of chloroplast ultrastructure

**Fig. 5:**
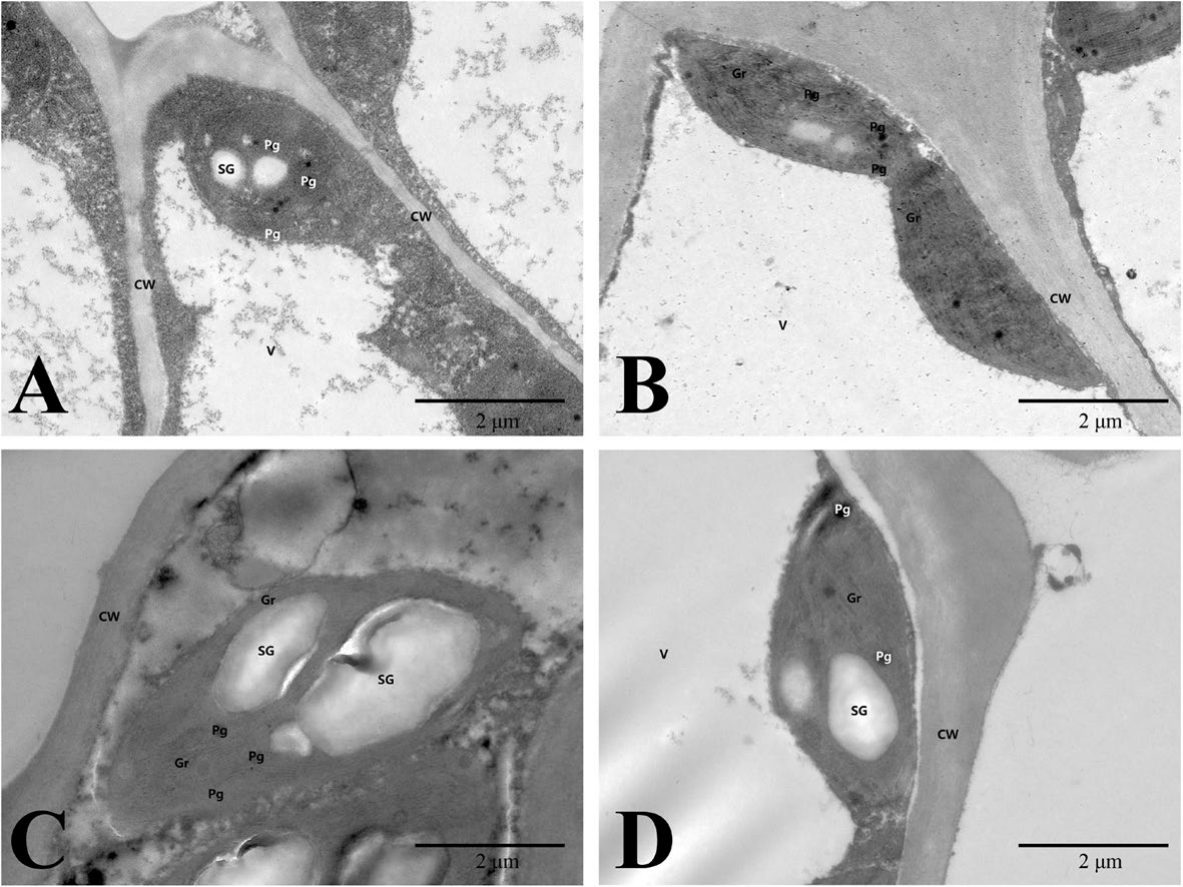
Chloroplast ultrastructures in leaves of ‘Ziyan Gongzhu’ at the four developmental stages. **A **T1, purple red leaf (RHS 67A); **B **T2 light purple leaf (RHS 65A); **C **T3, yellow green leaf (RHS 1C); **D **T4, dark green leaf (RHS N137A). CW: Cell wall; Gr: Grana; Pg: Plastoglobuli; SG: starch grain; V: vacuole. Bar = 2 µm

**Fig. 6:**
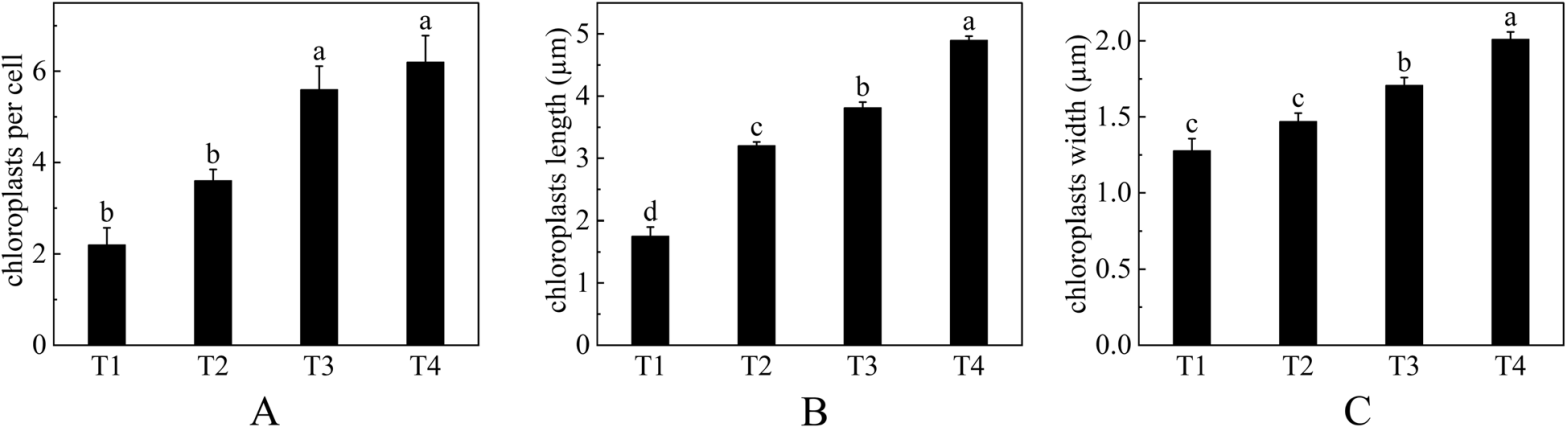
Average number of chloroplasts per cell (**A**), chloroplasts length (**B**) and width (**C**) in leaves of ‘Ziyan Gongzhu’ at the four developmental stages. Bars represent the standard errors of three biological replicates. Different lowercase letters indicate a significant difference (*p* < 0.05) relative to the value at the T4, as determined using ANOVA analysis, which is based on Duncan’s multiple range test. (T1: purple red leaf (RHS 67A), T2: light purple leaf (RHS 65A), T3: yellow green leaf (RHS 1C), T4: dark green leaf (RHS N137A)

Throughout the process of ‘Ziyan Gongzhu’ leaf color change, the shape and distribution of chloroplasts were compared. At the T1, the number of chloroplasts is few, there is no obvious grana lamellar structure, the cyst body in the matrix expands, and some plastoglobuli exist (Fig. 5A). At the T2, the number of chloroplasts is small, the structure of grana lamella is fuzzy and disordered, the stroma thylakoids are continuous, the number of plastid bodies is small and scattered, and a small amount of starch grains (SG) are visible (Fig. 5B). However, at the T3, the number of chloroplasts is moderate, grana thylakoids are arranged orderly, and a few plastoglobuli structures can be seen (Fig. 5C). Additionally, at the T4, the number of chloroplasts is relatively rich, the structure of grana thylakoid is clear, the cyst in the matrix is not significantly expanded (Fig. 5D). Much more chloroplasts were seen in T3, T4 leaves than in T1, T2 leaves (Fig. 6A). There were considerable changes in chloroplast length and breadth across leaves of various hues at four developmental phases (Fig. 6B, C). The studies demonstrated that variations in chloroplast structure affected leaf color.

### Analysis of chlorophyll fluorescence parameters

**Fig. 7:**
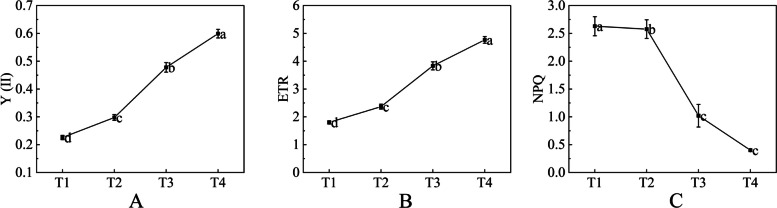
Chlorophyll fluorescence parameter Y(II) (reflects the actual photosynthetic efficiency, **A**), ETR (electron transfer rate, **B**) and NPQ (non-photochemical quenching, **C**) in leaves of ‘Ziyan Gongzhu’ at the four developmental stages. Bars represent the standard errors of three biological replicates. Different lowercase letters indicate a significant difference (*p* < 0.05) relative to the value at the T4, as determined using ANOVA analysis, which is based on Duncan’s multiple range test. (T1: purple red leaf (RHS 67A), T2: light purple leaf (RHS 65A), T3: yellow green leaf (RHS 1C), T4: dark green leaf (RHS N137A)

Y(II), The actual primary light capture efficiency when the PSII reaction center is partially closed under light conditions, which reflects the actual photosynthetic efficiency of plants. ETR, refers to the apparent electron transfer rate. Non-photochemical quenching (NPQ), fluorescence quenching caused by heat dissipation, reflects the photo-protection ability of plants [23]. In this study, the chlorophyll fluorescence parameters of ‘Ziyan Gongzhu’ leaves of different colors in four stages are shown in Fig. 7. The results show that the levels of Y(II) and ETR of T1, T2 and T3 were significantly lower than T4. Conversely, NPQ level in T1 and T2 were significantly higher than T3 and T4.

### RNA-Seq and de novo assembly

As shown in Table S3, the number of raw reads, clean reads and clean bases obtained from each library ranged from 37,766,474 to 50,998,826, 37,629,700–50829220 and 5.6–7.6G, respectively. The higher percentage of Q30 (95.30–96.15%), Q20 (98.54–98.85%) and clean reads (99.61–99.69%), and the lower quality sequences (0.22–0.28%), and reads containing poly-N (0.00%) and adaptor (0.08–0.11%) implied that the quality of RNA-Seq data was high and suitable for further analysis. Here, 87.01–87.77% (5.82–6.23%) of the clean reads were mapped uniquely (multiply) to *O. fragrans* genome (Table S4). A total of 45, 542 known (Table S5) and 2897 novel genes (Table S6) were identified in ‘Ziyan Gongzhu’ leaves.

### Functional annotation and DEGs

**Fig. 8:**
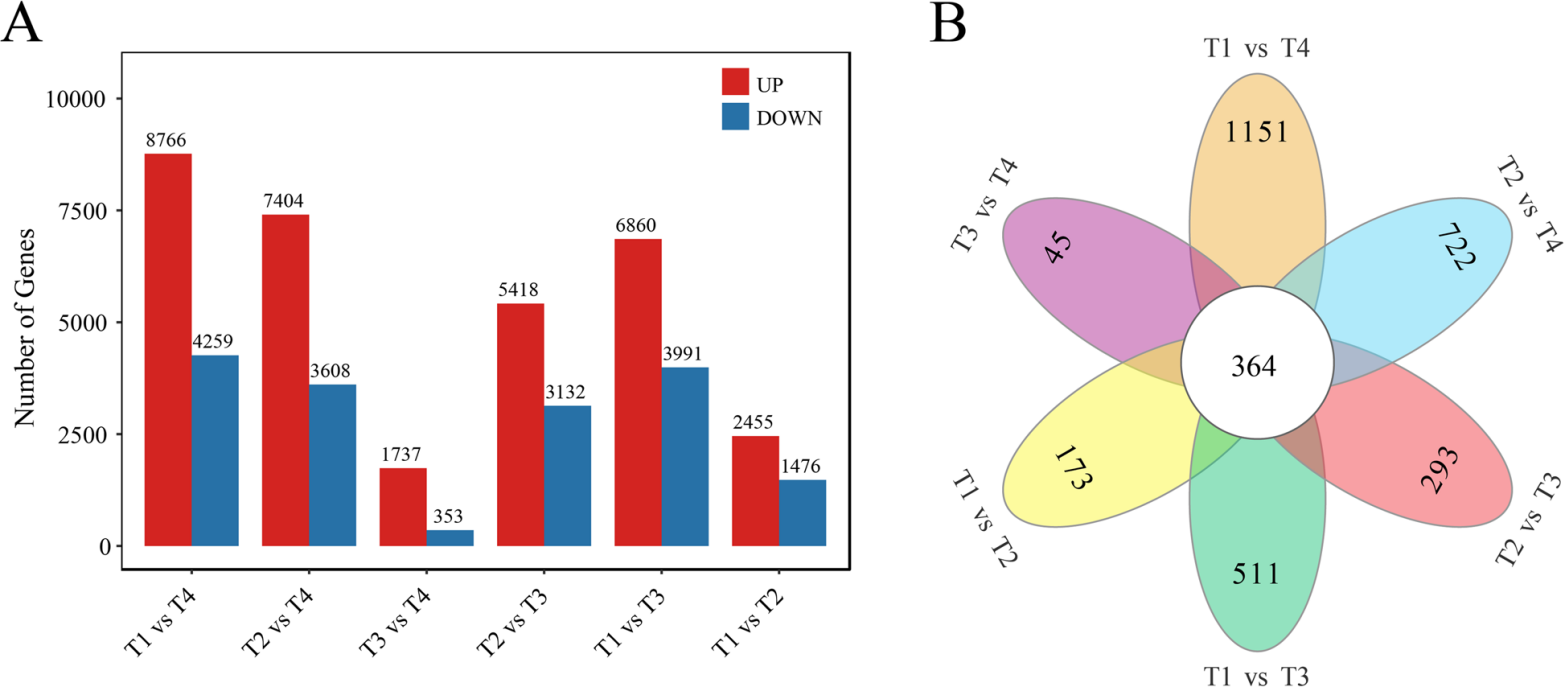
Upregulated and downregulated genes (**A**) and venn analysis of DEGs (**B**) identified in T1 vs T2, T1 vs T3, T1 vs T4, T2 vs T3, T2 vs T4 and T3 vs T4 of ‘Ziyan Gongzhu’. (T1: purple red leaf (RHS 67A), T2: light purple leaf (RHS 65A), T3: yellow green leaf (RHS 1C), T4: dark green leaf (RHS N137A)

As shown in Fig. 8A, we identified 1476 downregulated and 2455 upregulated, 6860 upregulated and 3991 downregulated, 8766 upregulated and 4259 downregulated, 5418 upregulated and 3132 downregulated, 7404 upregulated and 3608 downregulated, and 1737 upregulated and 353 downregulated genes in T1 vs T2, T1 vs T3, T1 vs T4, T2 vs T3, T2 vs T4 and T3 vs T4, respectively. For all DEGs identified here 173, 511, 1151, 293, 722 and 45 DEGs were identified only in T1 vs T2, T1 vs T3, T1 vs T4, T2 vs T3, T2 vs T4 and T3 vs T4, respectively, which comprised up 4.40%, 4.71%, 8.84%, 3.43%, 6.56% and 2.15% of DEGs identified in their corresponding comparative group, respectively (Fig. 8B).

We used KEGG pathway and GO enrichment analyses to get a deeper understanding of major DEG functions throughout changing leaf colors. In T1 versus T2, 312, 1746 and 489 DEGs had been significantly enriched to 132 GO terms in cellular component (CC), with 16 significant ones at adjusted *P* < 0.05, 382 in molecular function (MF), including 26 significant ones, and 730 in biological process (BP). 784, 4801 and 1272 DEGs between T1 and T3 were considerably enriched for 211 GO terms in CC, with 17 enriched ones, 568 in MF with 31 significant ones, and 1085 in BP with 142 significant ones. 936, 5741, and 1531 DEGs were enriched to 220 GO terms in CC with 25 considerable ones, 625 in MF with 29 significant ones, and 1158 in BP with 165 significant ones, in T1 vs T4. In T2 vs T3, 549, 3759, and 998 DEGs were enriched to 175 GO terms in CC with seven considerable ones, 511 in MF with 37 significant ones, and 979 in BP with 41 significant ones. For T2 vs T4, 753, 4842, and 1289 DEGs were enriched for 208 GO terms in CC with 17 significant ones, 583 in MF with 34 considerable ones, and 1062 in BP with 155 significant ones. In T3 vs T4, 142, 1004, and 282 DEGs were enriched to 97, 1004, and 282 GO terms in CC, MF, and BP, respectively, with 8, 30, and 556 significant ones, respectively (Fig. S1).

As shown in and Table S7, 651, 1911, 2358, 1500, 1967 and 396 DEGs were assigned to 117 KEGG pathways including 23 significantly enriched KEGG pathway with an adjusted *P* < 0.05, 134 KEGG pathway with 33 significantly enriched KEGG pathway, 132 KEGG pathway with 37 significantly enriched KEGG pathway, 127 KEGG pathway with 37 significantly enriched KEGG pathway, 132 KEGG pathway with 40 significantly enriched KEGG pathway and 105 KEGG pathways with 24 significantly enriched KEGG pathway in T1 vs T2, T1 vs T3, T1 vs T4, T2 vs T3, T2 vs T4 and T3 vs T4, respectively. Furthermore, the top 20 KEGG pathways with high representation from T1 vs T2, T1 vs T3, T1 vs T4, T2 vs T3, T2 vs T4 and T3 vs T4 comparisons were identified. The KEGG terms associated with pigment metabolism, such as phenylpropanoid biosynthesis (ko00940) and flavonoid biosynthesis (ko00941) were enriched in the T1 vs T2, T1 vs T4 and T2 vs T4 comparison, phenylpropanoid biosynthesis was enriched in the T2 vs T3 comparison, whereas in the T1 vs T3 comparison, the main enriched pigment metabolism pathways were phenylpropanoid biosynthesis and isoflavonoid biosynthesis (ko00943). In addition, phenylpropanoid biosynthesis, anthocyanin biosynthesis (ko00942) and carotenoid biosynthesis (ko00906) were enriched in the T3 vs T4 comparison.

### Phylogenetic analysis of *OfMYBs*

**Fig. 9:**
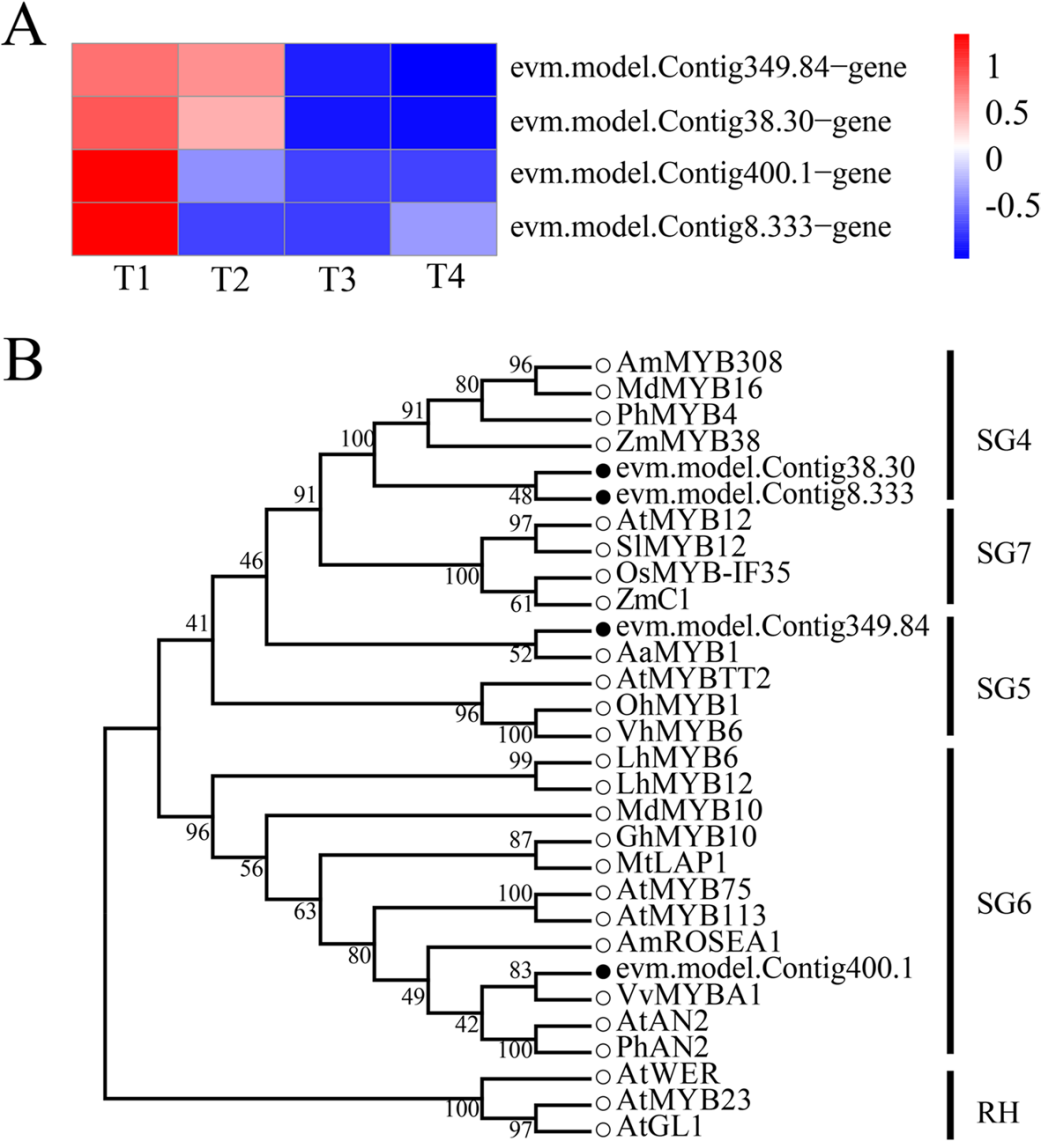
**A** Expression profiles of four differentially expressed *OfMYBs* at the four stages. **B** Phylogenic analysis of four *OfMYBs* with 26 flavonoid-related *MYBs* from other species. Full-length amino acid sequences from theseMYBs were analyzed under maximum-likelihood (ML) phylogenetic methods. Numbers near branches indicate bootstrap values that were calculated from 1,000 replicates. OfMYBs are highlighted with solid black circles. MYBs phylogenetic tree contained five subgroups (SGs), SG4 (repressors of flavonoid biosynthesis), SG5 (activators of anthocyanin biosynthesis), SG6 (activators of anthocyanin biosynthesis), SG7 (activators of flavonol/flavone biosynthesis), and RH (activators of root hair growth). The subgroup of RH was included as an outgroup. (T1: purple red leaf (RHS 67A), T2: light purple leaf (RHS 65A), T3: yellow green leaf (RHS 1C), T4: dark green leaf (RHS N137A)

Structural gene temporal and spatial expressions in the anthocyanin biosynthesis pathway, including DFR, F3H, and ANS, is generally regulated by transcription factors from bHLH, MYB, and WD40 [24]. On the basis of gene expression studies of *OfMYBs* and their evolutionary connections with 26 MYBs from other species implicated in flavonoid content [25–29], we identified four *OfMYBs* that may play critical functions for controlling flavonoid biosynthesis among the purple-red “Ziyan Gongshu” leaves. In the T1 stage, these *OfMYB* transcripts were most abundant (Fig. 9A and Table S8). The MYBs were grouped with cognizable functional homologs from SG4, SG5, and SG6 (Fig. 9B). In the SG4 group, *OfMYB1* and *OfMYB2* were closely related to *ZmMYB38*. In the SG5 group, *OfMYB3* clustered together with *AaMYB1*. In addition, *OfMYB4* was phylogenetically related to *VvMYBA1* from the SG6 group.

### Validation of RT-PCR

**Fig. 10:**
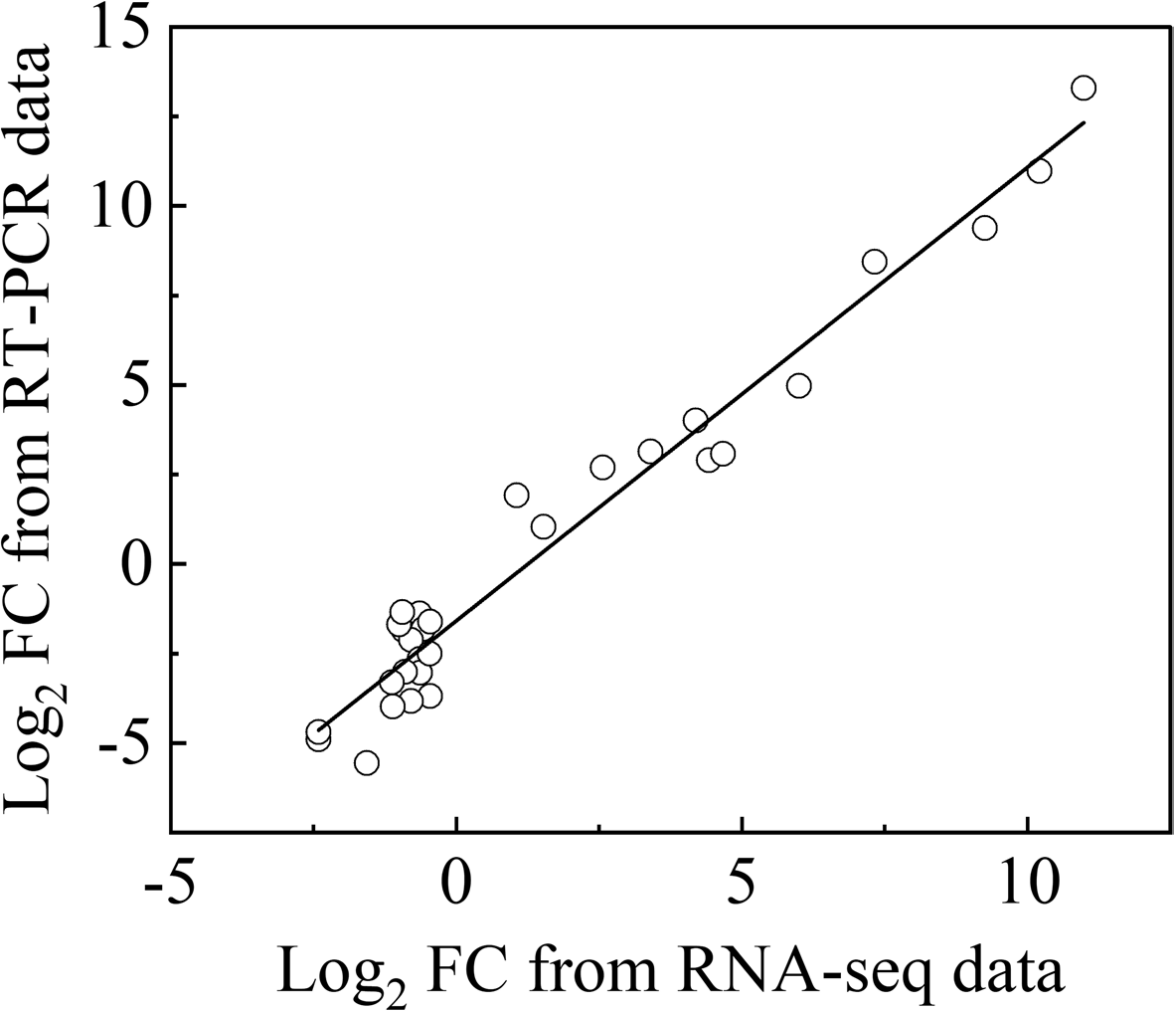
Correlation analysis of RNA-seq and RT-PCR data. DEGs were selected from the flavonoid and chlorophyll metabolic pathways. Expression levels obtained from the RT-PCR were normalized to the reference gene *Actin*. FC, Fold Change

The results of all DEGs obtained by RT-PCR analysis were highly related to data produced by RNA-Seq (Fig. 10). Thus, the RNA-Seq data were reliable.

### Analysis of metabolome of the leaves at different periods

T1, T2, T3, T4 leaf samples of *Osmanthus fragrans* ‘Ziyan Gongzhu’ were used for untargeted metabolome analysis. For positive (POS) and negative (NEG) ion modes, PCA revealed minimal metabolome variations among replicate samples from same periods but significant ones among those from distinct periods, indicating that data reproducibility was high and the experiment was trustworthy (Fig. S2). They were appropriate for further qualitative and quantitative examination. A hierarchical heatmap clustering analysis of the samples was conducted using the metabolite concentration data from POS and NEG (Fig. S3). Based on the findings of a correlation study employing POS and NEG samples, the metabolite makeup of the four substances varied substantially (Fig. S4).

### Identification of metabolites of *Osmanthus fragrans* ‘Ziyan Gongzhu’ leaves

**Fig. 11:**
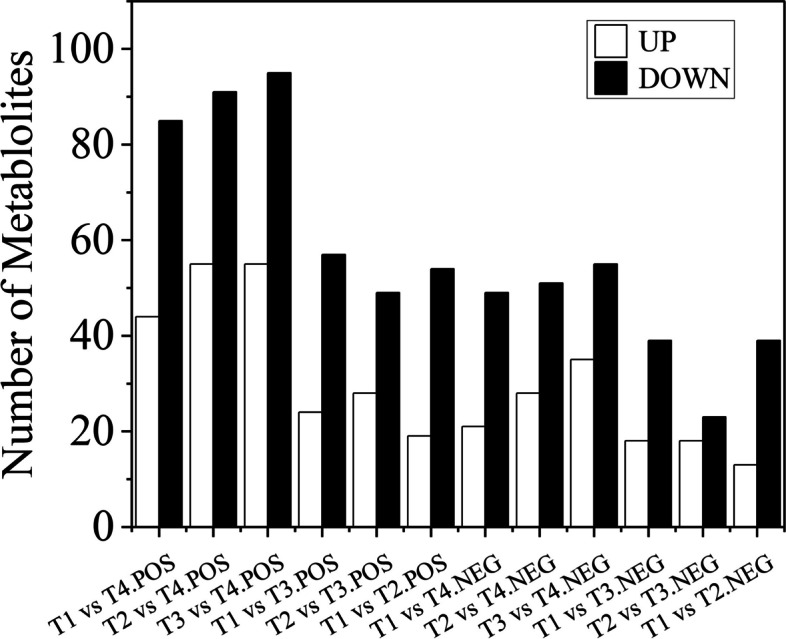
Upregulated and downregulated metabolites in T1 vs T2, T1 vs T3, T1 vs T4, T2 vs T3, T2 vs T4 and T3 vs T4 of ‘Ziyan Gongzhu’. (T1: purple red leaf (RHS 67A), T2: light purple leaf (RHS 65A), T3: yellow green leaf (RHS 1C), T4: dark green leaf (RHS N137A)

**Fig. 12:**
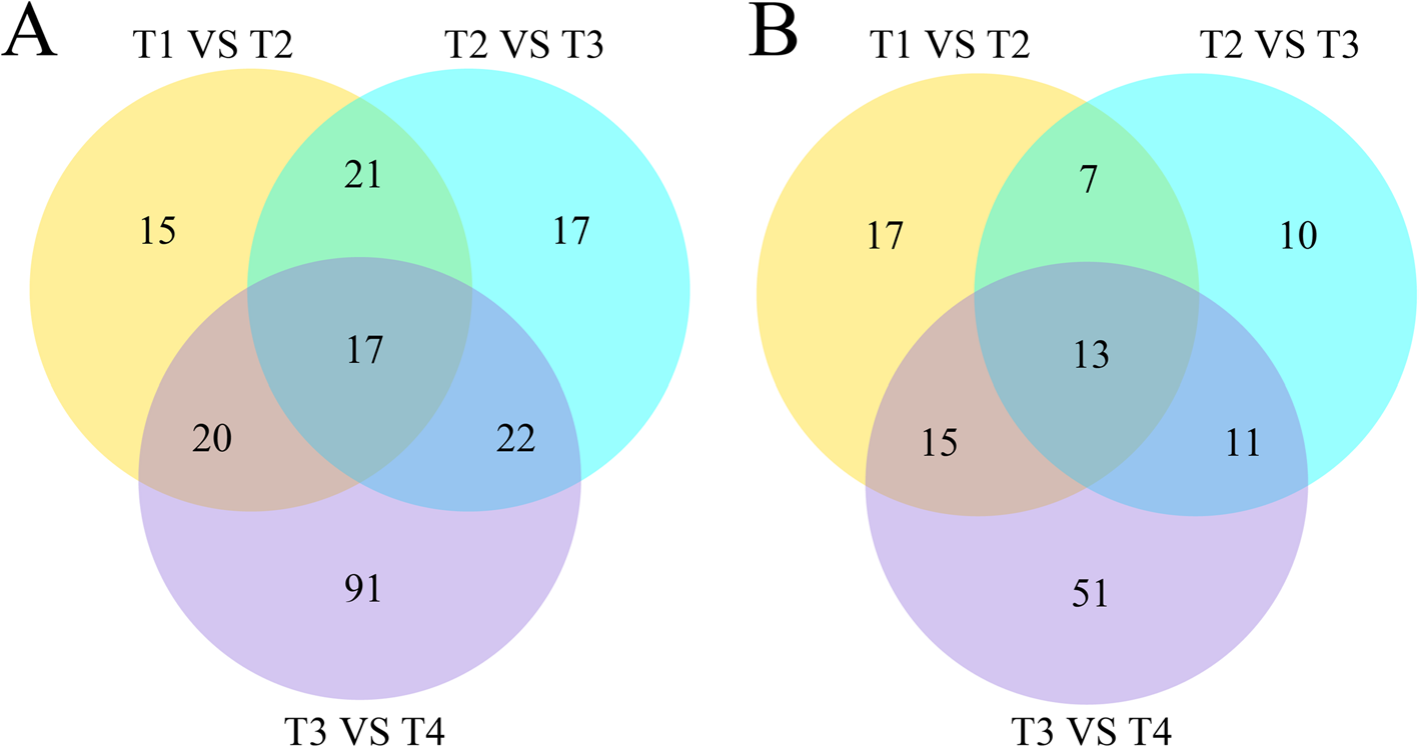
Venn analysis of DAMs identified in T1 vs T2, T1 vs T3, T1 vs T4, T2 vs T3, T2 vs T4 and T3 vs T4 of ‘Ziyan Gongzhu’ in POS (**A**) and NEG (**B**). (T1: purple red leaf (RHS 67A), T2: light purple leaf (RHS 65A), T3: yellow green leaf (RHS 1C), T4: dark green leaf (RHS N137A)

The untargeted metabolome analysis of the four POS and NEG samples showed 1489 and 901 unique compounds, respectively. In POS, all 1489 components detected were classified into twelve categories, including 106 lipids, 86 organic acids, 58 flavonoids, 36 nucleotides and derivatives, 32 organooxygen compounds, 22 lignans and coumarins, 17 indoles and derivatives, 12 esters, 5 alkaloids, 4 carbohydrates and carbohydrate conjugates and 1111 other types; In NEG, all 901 components detected were classified into eleven categories, including 59 lipids, 34 organooxygen compounds, 32 flavonoids, 31 organic acids, 21 nucleotides and derivatives, 7 esters, 4 lignans and coumarins, 4 indoles and derivatives, 4 alkaloids, 4 carbohydrates and carbohydrate conjugates and 701 other types (Table S9). The differential metabolite analysis results showed that T3 vs T4 had the most differential metabolites of 150 and 90 in POS and NEG, respectively, of which 55 were downregulated and 35 upregulated in NEG, and 95 were downregulated and 55 upregulated in POS (Fig. 11). In NEG, 52, 57, 70, 41 and 79 differential metabolites were observed for T1 vs T2, T1 vs T3, T1 vs T4, T2 vs T3 and T2 vs T4, respectively; In POS, 73, 81, 129, 77 and 146 differential metabolites were observed for T1 vs T2, T1 vs T3, T1 vs T4, T2 vs T3 and T2 vs T4, respectively. The link between groups and distinct metabolites is seen in Fig. 12. As seen in the picture, the T3 vs. T4 combination contained the most distinct metabolites, with 91 and 51 in POS and NEG, respectively. In POS, T1 vs. T2 and T3 vs. T4 combinations shared the greatest number of divergent metabolites, totaling 39; in NEG, T2 vs. T3 and T3 vs. T4 combinations shared the greatest number of differential metabolites, totaling 28.

All DAMs were submitted to KEGG database for functional annotation (Fig. S5 and Table S10). A total of 34, 37, 48, 26, 54 and 52 DAMs were annotated to 58, 57, 46, 35, 54 and 57 KEGG pathways in T1 vs T2, T1 vs T3, T1 vs T4, T2 vs T3, T2 vs T4 and T3 vs T4, respectively. Phenylpropanoid biosynthesis (ko00940) was the only 1 significantly enriched KEGG pathway for DAMs in T2 vs T3 at Q-value < 0.05. The KEGG terms associated with pigment metabolism, such as anthocyanin biosynthesis (ko00942), phenylpropanoid biosynthesis (ko00940), flavonoid biosynthesis (ko00941), Porphyrin and chlorophyll metabolism (ko00860) were enriched in the T1 vs T2 comparison. In addition, anthocyanin biosynthesis, phenylpropanoid biosynthesis, flavonoid biosynthesis was enriched in the T1 vs T3, T1 vs T4, T2 vs T3, T2 vs T4 and T3 vs T4 comparison.

### Conjoint analysis of transcriptomic and metabolomic data

We detected much more KEGG-enriched DEG pathways than DAM pathways. There were 51, 53, 43, 29, 50 and 45 common KEGG pathways between DAMs and DEGs in T1 vs T2, T1 vs T3, T1 vs T4, T2 vs T3, T2 vs T4 and T3 vs T4, respectively (Table S11). As shown in Table S11, we obtained 28, 30, 22, 16, 24 and 22 enriched KEGG pathways with a *P* < 0.05 for DEGs and/or DAMs in T1 vs T2, T1 vs T3, T1 vs T4, T2 vs T3, T2 vs T4 and T3 vs T4, respectively. There were 6, 4, 3, 3, 2 and 2 common enriched KEGG pathways between DEGs and DAMs in T1 vs T2, T1 vs T3, T1 vs T4, T2 vs T3, T2 vs T4 and T3 vs T4, respectively.

**Fig. 13:**
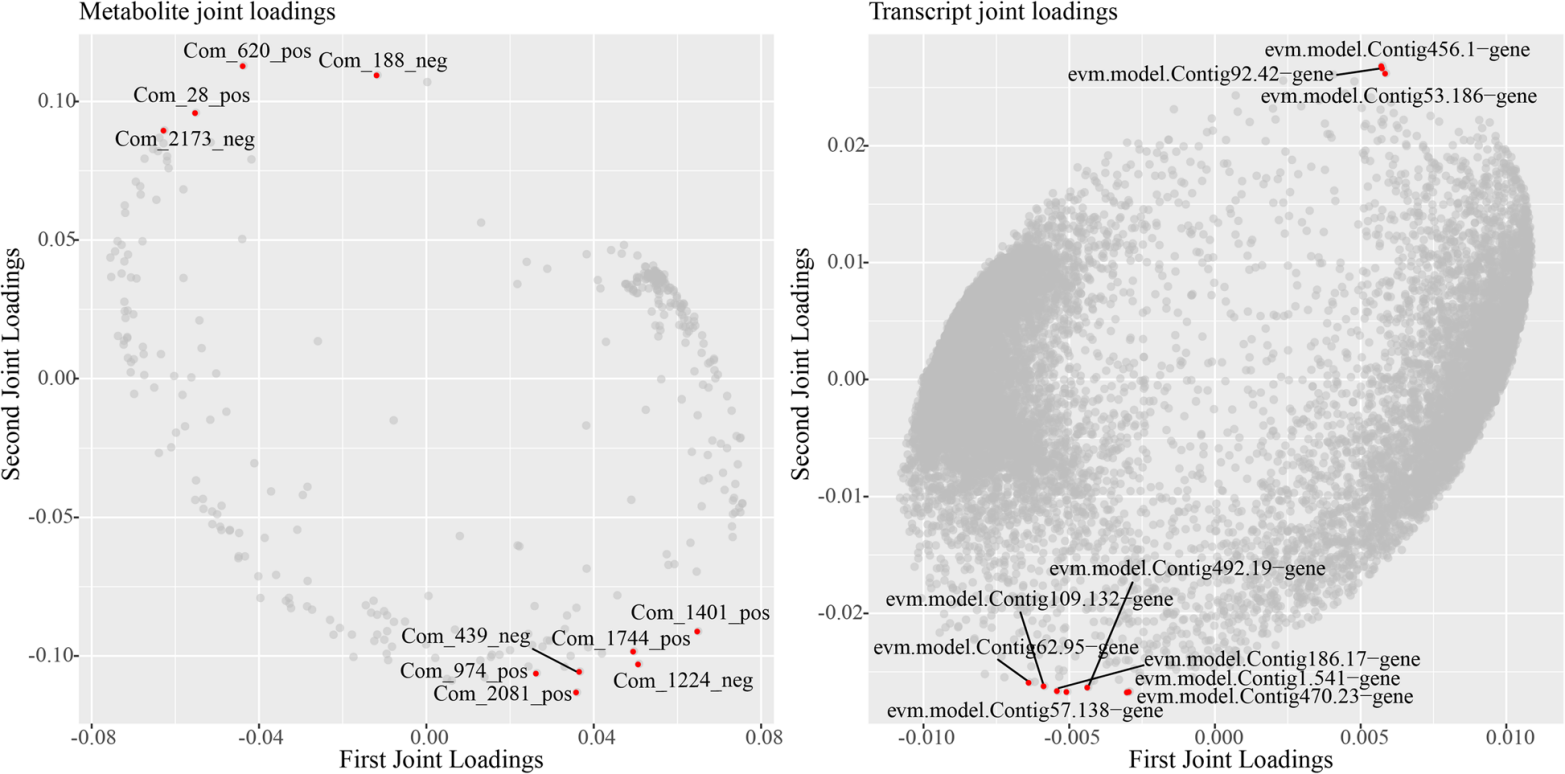
Two-way Orthogonal Partial Least Square (O2PLS) loading plots for DAMs (A) and DEGs (B). The top 10 DEGs and DAMs that had greater influence on the other omics were indicated by red dots

O2PLS model was used to integrate the transcriptomic and metabonomic datasets (Fig. 13). evm.model.Contig456.1-gene, evm.model.Contig92.42-gene, evm.model.Contig53.186-gene, evm.model.Contig.

62.95-gene, evm.model.Contig109.132-gene, evm.model.Contig1.541-gene, evm.model.Contig186.17-gene, evm. model.Contig57.138-gene, evm.model.Contig470.23-gene and evm.model.Contig492.19-gene (Com_2173_neg, Com_28_pos, Com_620_pos, Com_188_neg, Com_974_pos, Com_1744_pos, Com_1401_pos, Com_1224_neg, Com_439_neg and Com_2081_pos) were the top 10 DEGs (DAMs) that had greater influence on the other omics.

## Discussion

### Flavonoid biosynthesis played a role in color mechanism of *Osmanthus fragrans* ‘Ziyan Gongzhu’ leaves

In previous studies, the colorful colors of colored-leaf plants are mainly due to the changes in the types, contents and distribution of pigments in leaf cells [30, 31]. The anthocyanin content in T1 was the highest, 7.19 mg/g, and the leaves were purple red; the anthocyanin content in T3 was the lowest, 2.27 mg/g (Table 1). At T4, the leaves are dark green. The increase of anthocyanin content at T4 did not necessarily show color, which may be related to the arrangement and distribution of pigments in leaves.

**Fig. 14:**
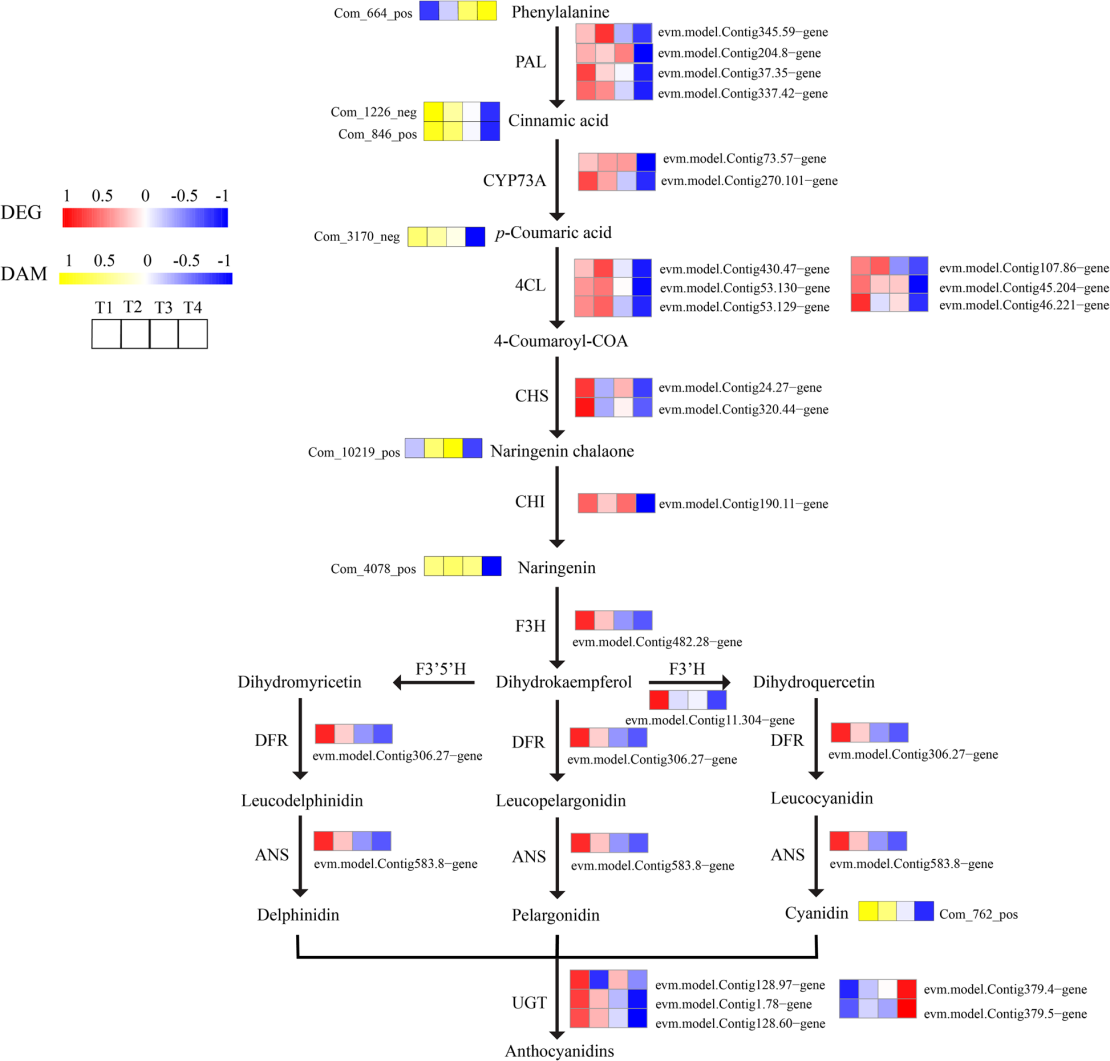
Expression profiles of the DEGs and accumulation of the DAMs involved in flavonoid biosynthesis pathway in leaves of ‘Ziyan Gongzhu’ at the four developmental stages. The scale bar represents the changes of gene expression pattern/metabolite accumulation: red/yellow rectangle indicates upregulated expression pattern, blue rectangle indicates downregulated expression pattern, and the white rectangle indicates gene or metabolite whose expression and accumulation did not change. The normalized signal intensity ranged from -1.0 to 1.0, which was consistent with color changes from blue to red/yellow. (T1: purple red leaf (RHS 67A), T2: light purple leaf (RHS 65A), T3: yellow green leaf (RHS 1C), T4: dark green leaf (RHS N137A)

As anticipated, the higher anthocyanin content of T1 purple-red leaves was associated with the increased expression of numerous structural genes implicated in the flavonoid pathway. We discovered 23 DEGs along this route (Fig. 14). Among these are many DEGs implicated in the early flavonoid production pathway, such as CHS, CHI, F3H, F3’H, DFR, ANS and UGT showed the highest expression levels at the T1 in ‘Ziyan Gongzhu’ leaves. Also, we identified 1 upregulated Phenylalanine, 2 downregulated cinnamic acid, 1 downregulated p-coumaric acid, 1 downregulated naringenin and 1 downregulated cyanidin from T1 to T4 in ‘Ziyan Gongzhu’ leaves (Table S12).

We obtained 4 PAL, 2 CYP73A (C4H), 6 4CL, 2 CHS, 1 CHI in ‘Ziyan Gongzhu’ leaves, all of which were highly expressed from T1 to T3 and lowest in T4. Following light induction, the PAL activity and anthocyanin concentration of purple-foliage plum leaves increased. The leaves progressively took on a purplish-red hue [32]. The regulation of C4H and 4CL activity is closely related to anthocyanin accumulation and leaf color change. CHS is a crucial enzyme in the production route for anthocyanins. By suppressing the expression of *Torenia hybrida CHS* through RNA interference [33], blue *T. hybrida*, which is rich in mallow pigment and peony pigment, may be converted into white *T. hybrida* with anthocyanin insufficiency. The silencing of *CHI* may also cause tobacco and carnation to become yellow [34]. However, we found that PAL, CYP73A (C4H), 4CL, CHS, CHI in the flavonoid pathway were expressed at the lowest level at the T4 and expressed highly level from T1 to T3 (Fig. 14). These genes not only regulate the synthesis of anthocyanins, but also are closely related to the synthesis of lignin and flavonoids [35]. Similarly, we obtained 1 upregulated phenylalanine, 2 downregulated cinnamic acid, 1 downregulated p-coumaric acid and 1 downregulated naringenin, which as the precursor of various flavonoids participate in the final formation of downstream secondary metabolites [33]. Wei et al. (2016) [36] got a similar result. Thus, PAL, CYP73A (C4H), 4CL, CHS and CHI genes might not be critical genes affecting purple red coloration in ‘Ziyan Gongzhu’ leaves. We also found that 1F3H, 1 F3’H, 1 DFR, 1 ANS and 3 UGT in this pathway were expressed at the highest level at the T1. Jiang et al. (2013) [37] showed that antisense inhibition of *F3H* expression in strawberry (*Fragaria* × *ananassa*) would block anthocyanin synthesis, thus affecting anthocyanin accumulation in fruit. *DFR* in *Cymbidium hybrida* cannot reduce dihydrokaempferol, resulting in a lack of pelargonium. Therefore, it may be necessary to transform DFR genes that can effectively catalyze DHK reduction to develop orange hybrid *Cymbidium hybrida* [38]. When the ANS gene of onion is mutated, the peel color will change from purple to yellow [39]. Lee et al. (2017) [40] revealed that overexpression of *AtUGT* in transgenic plants enhanced anthocyanin synthesis efficiently. In addition, 1 F3H, 1 DFR, 1 ANS and 3 UGT were strongly expressed at T1, indicating that their expression might accelerate the production of stable anthocyanins in the purple-red leaves of ‘Ziyan Gongzhu’. In addition, the non-targeted metabolome detected that cyanidin was gradually downregulated from T1 to T4, indicating that the pigments closely related to the color leaves of ‘Ziyan Gongzhu’ were cyanidin rather than delphinidin and pelargonidin. In the present study, the highest content of cyanidin at T1 was detected in the non-targeted metabolic, and F3H, F3’H, DFR, and ANS were highly expressed at the T1, suggesting that cyanidin may be responsible for the purple-red hue of ‘Ziyan Gongzhu’ leaves, and F3H, F3'H, DFR, and ANS regulate cyanidin accumulation throughout this process.

In this study, we screened four *OfMYBs* whose expression levels were highest at the T1 (Fig. 9A). Further analysis showed that these genes clustered together with MYBs related to flavonoid biosynthesis in other species (Fig. 9B). Among them, *OfMYB1 and OfMYB2* are closely related to *ZmMYB38*. *ZmMYB38* is a known regulator of the flavonoid pathway, and can directly bind to the ZmCOMT1 gene [41]. *PhMYB4* [42] also has been demonstrated to possess strong repressive effects on flavonoid pathway genes. In contrast, two potential anthocyanin-related MYB activators, *OfMYB3* (a homolog of *AaMYB1*) and *OfMYB4* (homologs of *VvMYBA1*), were also identified. In previous studies, *AaMYB1* from anthurium (*Anthurium andraeanum*) was shown to be capable of activating anthocyanin production in the petals of an orchid (*Cymbidium spp.*) cultivar [43]. *VvMYBA1* gene plays an important role in regulation of anthocyanin biosynthesis in grapes [44]. Hence, these homologous *OfMYBs* may play crucial roles in the coloring of the purple-red leaves of ‘Ziyan Gongzhu’. Taken together, these results suggested that flavonoids accumulation in ‘Ziyan Gongzhu’ red leaves may be controlled by a set of *OfMYB* genes, which includes at least two putative activators (*OfMYB3* and *OfMYB4*) and two putative repressors (*OfMYB1* and *OfMYB2*). Future study will investigate the biological activities of these essential *OfMYBs*, which may disclose how these genes control flavonoid production in ‘Ziyan Gongzhu’ leaves.

### Chloroplast development and regulation of Chl-related genes in ‘Ziyan Gongzhu’ leaves

The abnormal development of chloroplast affects the content of chlorophyll and also leads to abnormal leaf color. The process of chloroplast differentiation and development can be divided into seven steps: nuclear gene transcription, chloroplast protein input and processing, chloroplast gene transcription and translation, thylakoid formation, pigment synthesis, plasmid–nuclear signal transduction, and chloroplast division [45]. The variation of leaf color of color leafed plants is closely related to the abnormal development of chloroplasts. For example, Li et al. (2018) [46] found in the study of *ginkgo biloba* that the chloroplast of the gold leaf mutant is smaller than that of the normal green leaf plant, and the chloroplast structure of the mutant has also undergone significant changes, such as thylakoid membrane rupture, blurred matrix lamella, irregular vesicle arrangement, etc. A study shows that changes in thylakoid and vesicle structures in *Arabidopsis* mutants can lead to the formation of *‘vipp1’,* which exhibiting pale cotyledons and green true leaves [47]. In the T1, T2, and T3 leaves, the chloroplasts lacked an entire inner membrane structure, the grana lamella structure was fuzzy and disorganized, and the stroma thylakoid enlarged. In addition, the quantity of chloroplasts reduced relative to the T4 stage, showing that chloroplast formation was aberrant throughout these phases (Fig. 5). Much more chloroplasts were seen in T3, T4 leaves than in T1, T2 leaves (Fig. 6A). There were considerable changes in chloroplast length and breadth across leaves of various hues at four developmental phases (Fig. 6B, C). Hence, we predicted that aberrant chloroplasts may impact the formation of the purple, red, light purple, and yellow-green leaves of “Ziyan Gongzhu,” therefore limiting Chl biosynthesis and leaf color diversity.

From T1 to T4, the contents of chlorophyll (Chl) a + b in leaves showed an overall upward trend. The chl a + b content in T1 was the lowest, 0.24 mg/g, and the chl a + b content in T4 was the highest, 1.99 mg/g (Table 1). Combined with the observation results of chloroplast ultrastructure, we speculate that the abnormal chloroplast development may affect the biosynthesis of Chlorophyll, resulting in the color leaves of ‘Ziyan Gongzhu’.

**Fig. 15:**
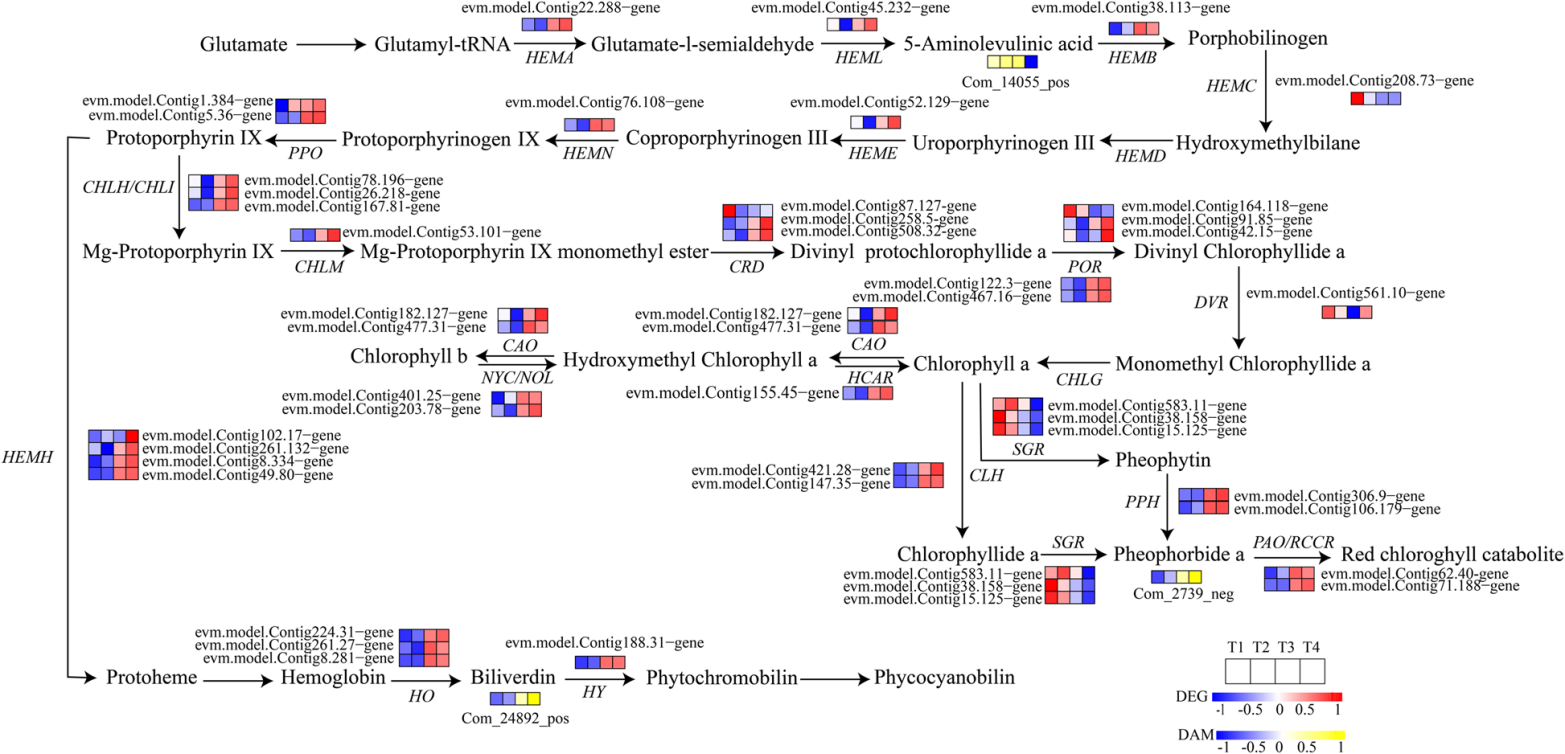
Expression profiles of the DEGs and accumulation of the DAMs involved in chlorophyll and heme metabolism pathway in leaves of ‘Ziyan Gongzhu’ at the four developmental stages. The scale bar represents the changes of gene expression pattern/metabolite accumulation: red/yellow rectangle indicates upregulated expression pattern, blue rectangle indicates downregulated expression pattern, and the white rectangle indicates gene or metabolite whose expression and accumulation did not change. The normalized signal intensity ranged from -1.0 to 1.0, which was consistent with color changes from blue to red/yellow. (T1: purple red leaf (RHS 67A), T2: light purple leaf (RHS 65A), T3: yellow green leaf (RHS 1C), T4: dark green leaf (RHS N137A)

In this study, 35 DEGs involved in Chl biosynthesis and degradation and 8 DEGs involved in Heme metabolism were identified according to KEGG pathway assignment, and the expression profiles of these DEGs were analyzed via hierarchical clustering (Fig. 15). The expression of most genes was upregulated during the gradual greening of leaves from T1 to T4. In addition, we identified 1 downregulated 5-Aminolevulinic acid, 1 upregulated pheophorbide a, 1 upregulated biliverdin from T1 to T4 in ‘Ziyan Gongzhu’ leaves (Table S13).

Firstly, we isolated one Glutamyl-tRNA reductase (HEMA) gene were expressed at the high level in the T3, T4, which is a key rate limiting enzyme for tetrapyrrole synthesis and a key enzyme for chlorophyll metabolism. Previous studies found that silencing *HEMA1* reduced chlorophyll content in *A. thaliana* [48]. Glutamyl-tRNA reductase (GluTR) and Glutamate-1-semialdehyde 2, 1-aminomutase (GSA) catalyze L-glutamyl-tRNA production δ- Aminolevulinic acid (ALA). ALA is an important precursor of chlorophyll synthesis, which controls the rate of chlorophyll and heme synthesis. we isolated one ALA with the lowest content in T4. Then, Coproporphyrinogen III oxidase (CPOX) and Protoporphyrinogen oxidase (PPOX) are also key enzymes in the process of plant chlorophyll biosynthesis, and the production of many plant chlorophyll loss mutants has been proved to be related to these two enzymes, such as *Brassica Juncea* [49]*, Bambusa multiplex* 'Silverstripe' R. A. Young [50]*, Dieffenbachia camile* [51]*, Euonymus japonicus* L. var. *aureamarginatu* and *E. japonicus* L. var. *aureovariegatus* [52] and so on*.* In this study, we isolated that one CPOX and two PPOX genes were expressed at the high level in the T3, T4. After that, protoporphyrin IX (Proto IX) is the watershed of tetrapyrrole biosynthesis pathway, one of which is the iron branch, and the other is the magnesium branch of Chl synthesis. Magnesium chelation reaction is the sign of Proto IX entering the chlorophyll synthesis pathway. At the same time, Magnesium chelatase (MgCh) is also one of the key enzymes of chlorophyll biosynthesis. Mg-protoporphyrin IX methyltransferase (MgPMT) and (Mg-protoporphyrin IX monomethyl ester) MgPEC are also important for chlorophyll synthesis. MgCh contains H (CHLH), I (CHLI) and D subunit (CHLD), and its function depends on the synergistic effect of these three functional subunits. We obtained 4 genes encoding MgCh (CHLH, CHLI, CHLM), 2 CRDs were expressed at the high level in the T3, T4 and 1 CRD was expressed at the high level in the T1 in ‘Ziyan Gongzhu’ leaves. Previous studies have shown that the single base mutation in the third exon of the *GUN5* gene encoding magnesium chelatase subunit H in *Arabidopsis* will lead to the formation of albinism [53]. And the homozygous mutant *chlm* of *Arabidopsis thaliana* shows the characteristics of dwarfism and albinism at the seedling stage, produces yellow leaflets without chlorophyll under weak light [54]. And when the antisense *CRD1* gene is transferred into *Arabidopsis*, the T_1_ transgenic *Arabidopsis* has early death, plant dwarfing, and leaves show a yellow phenotype during development [55]. Furthermore, we also found that 1 DVR, 2 CAO in this pathway were expressed at the high level in the T3, T4. 3,8-Divinyl protochlorophyllide a 8-vinyl reductase (DVR) is an essential key enzyme for the synthesis of normal MV-Chl in plants. The *824ys* mutant of rice lost 9 nucleotides in the ORF of the *OsDVR* gene, resulting in the loss of 3 amino acids in the coding protein, complete loss of enzyme activity, and extremely significant decrease in its chlorophyll content [56]. And Chlorophyll ester oxidase (CAO) is the key enzyme for controlling chlorophyll b to chlorophyll a. To sum up, the transcription level of most genes encoding key enzymes for chloroplast synthesis, such as HEMA, CAO, DVR, CRD, and the concentration of Chl in T1 and T2 are significantly lower than those in T3 and T4, and the content of one ALA is the least in T4. Therefore, our results show that the low expression of these genes at T1 and T2 reduces the enzyme activity, affects the chlorophyll synthesis, and may causes the purple red and red leaves of ‘Ziyan Gongzhu’ at T1 and T2, respectively, while the high transcription level of genes encoding key enzymes of chlorophyll synthesis in T3 and T4 increases the rate of chlorophyll synthesis, which may lead to yellow green and green leaves of T3 and T4, respectively.

Abnormal chlorophyll degradation pathway will also cause certain changes in plant leaf color. The degradation process of chlorophyll can be divided into two stages: chlorophyll is degraded into the primary fluorescent chlorophyll catabolite (pFCC) after four enzymatic steps; The reaction site is transferred to the vacuole, and the nonfluorescent chlorophyll catabolites (NCCs) are generated under the catalysis of the acid vacuole. These products are converted into pyrrole degradation products [57]. Chl b reductase (NOL and NYC1) is the key enzyme in the degradation process of chlorophyll a/b protein complex. Teng et al. (2021) showed that overexpression of *ZjNYC1* gene would accelerate the degradation of chlorophyll in *Arabidopsis*, promote senescence through the accumulation of ABA and reactive oxygen species (ROS), and negatively affect the integrity and functionality of the light system [58]. CLH is a rate limiting enzyme in the degradation of Chl. High expression of *CHL2* and *RCCR* encoding key chlorophyll degradation enzymes leads to yellow leaf *Cymbidium sinense* mutants [59]. Previous studies reported that the expression of PAO was negatively correlated with chlorophyll level. *PAO* is highly expressed in the yellow leaves of *Ficus carica*, indicating that it plays a key role in the process of chlorophyll degradation [60]. The discovery of SGR was a landmark in the study of plant chlorophyll degradation mechanisms. The expression of *SGR* in senescent leaves increases rapidly, which makes green leaves fade slightly in *Arabidopsis* [61]. The expression of *SGR* in yellow leaf Orchid varieties is higher than that in green leaf varieties [62]. In this study, we identified that 2 CLH, 2 NOL/NYC1, 2 PAO/RCCR were highly expressed in T3 and T4, which may lead to faster degradation of Chl, and a Pheophorbide a whose content increased from T1 to T4, resulting in the production of yellow green leaves in T3. However, we also screened 3 SGR genes with the lowest expression at T4, which may hinder the degradation of chlorophyll, leading to green leaves at T4. Although our results show that some genes related to chlorophyll degradation are highly expressed in T3 and T4 and the content of a chl decomposition metabolite Pheophorbide a was upregulated from T1 to T4, the chl content increases. We speculate that it may be due to the post transcriptional regulation of PAO and NOL/NYC and other genes that restrict the biological activity of enzymes, or the degradation rate of Chl is slower than its biological synthesis rate.

In plants, heme and chlorophyll share the same precursor, and chlorophyll production may be controlled by heme feedback inhibition. Consequently, disruption of the heme metabolic branches of plants inhibits the synthesis of ALA owing to negative feedback regulation, resulting in suppression of chlorophyll production and differences in leaf color [63]. Zhu et al. (2017) [64] found in a *Brassica napus* chlorophyll-deficient mutant *(ygl)* that the expression of *BnaA07. HO1* was down regulated, and the loss of *BnaC07.HO1* damaged the metabolism of tetrapyrrole, especially the biosynthesis of chlorophyll, resulting in yellow green leaves of seedlings. Both pea and Arabidopsis leaf color mutants are caused by HO1 deficiency [65]. In this study, we screened 3 HO, 1 HY genes with low expression in T1 and T2 but high transcription level in T3 and T4, and 1 Biliverdin with gradually increased content from T1 to t4. The low transcription level of the 3 HO genes in T1 and T2 may lead to the accumulation of heme, hinder the synthesis of chlorophyll and affect leaf color.

### Regulation of carotenoid-related genes in ‘Ziyan Gongzhu’ leaves

The content of carotenoids was the lowest at T2, 0.27 mg/g, and the highest at T4, 0.87 mg/g (Table 1). In conjunction with the structural properties of chloroplasts, our results indicated that chloroplast insufficiency not only suppressed Chlorophyll production in the colorful leaves of ‘Ziyan Gongzhu’ plants, but also carotenoid biosynthesis.

**Fig. 16:**
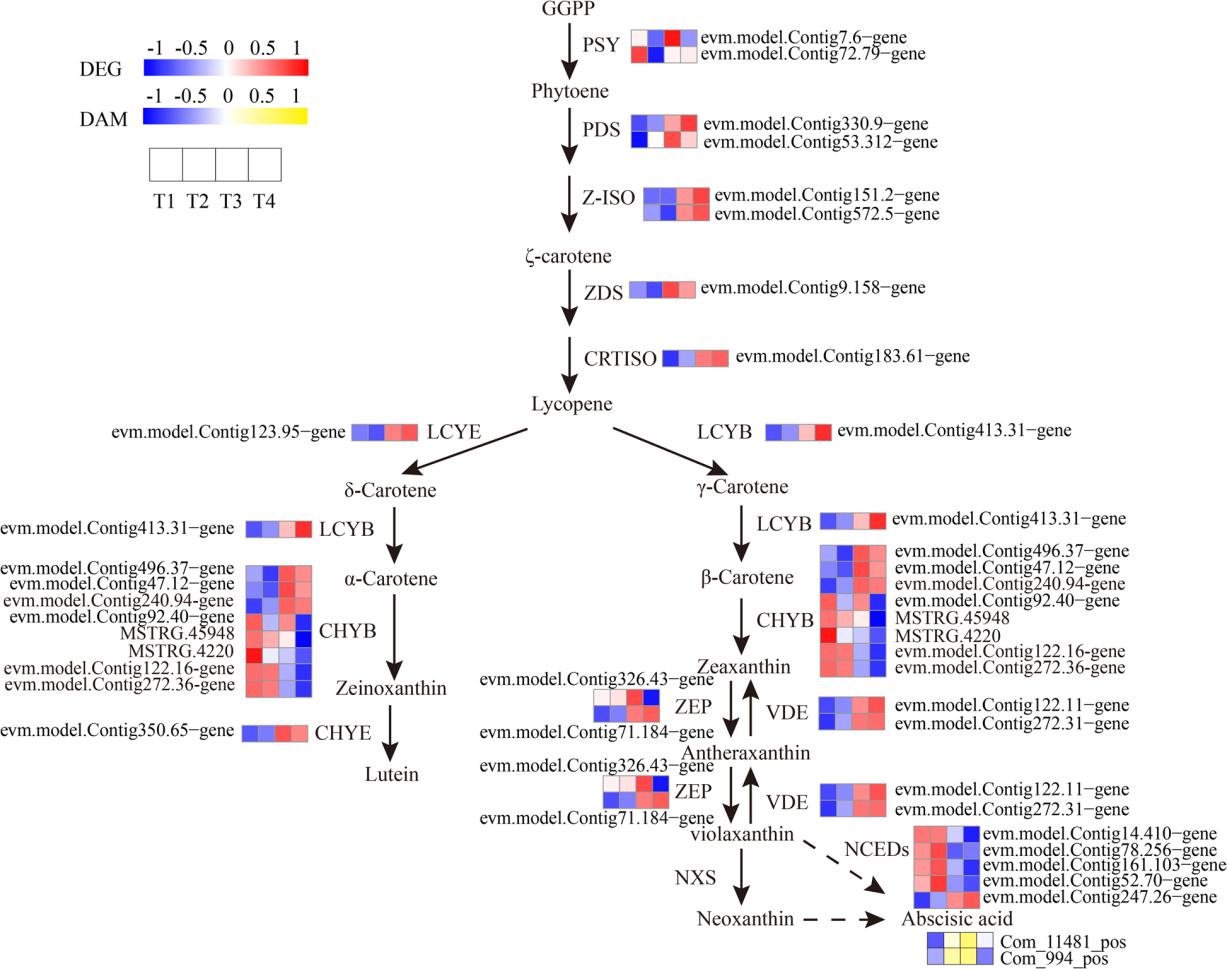
Expression profiles of the DEGs and accumulation of the DAMs involved in carotenoid metabolic pathway in leaves of ‘Ziyan Gongzhu’ at the four developmental stages. The scale bar represents the changes of gene expression pattern/metabolite accumulation: red/yellow rectangle indicates upregulated expression pattern, blue rectangle indicates downregulated expression pattern, and the white rectangle indicates gene or metabolite whose expression and accumulation did not change. The normalized signal intensity ranged from -1.0 to 1.0, which was consistent with color changes from blue to red/yellow. (T1: purple red leaf (RHS 67A), T2: light purple leaf (RHS 65A), T3: yellow green leaf (RHS 1C), T4: dark green leaf (RHS N137A)

We identified 28 DEGs in the carotenoid metabolic pathway, the abundance of their transcripts varied among the different stages. The expression levels of the DEGs involved in α-carotene and β-carotene biosynthesis, such as PDS, Z-ISO, ZDS, LCYB and LCYE, were much higher at the T3 or T4. Similarly, the most of CHYB (β-carotene hydroxylase) and NCED (9-cis-epoxycarotenoid dioxygenase), acting as the critical genes in zeinoxanthin, zeaxanthin and abscisic acid (ABA) biosynthesis, respectively, were highly expressed at the T1 or T2. Additionally, CHYE and VDE (violaxanthin de-epoxidase), which play important roles in the biosynthesis of lutein, antheraxanthin, and violaxanthin, were much higher at the T3 or T4 (Fig. 16 and Table S14). In addition, we identified 2 ABA were much higher at the T2 or T3 in ‘Ziyan Gongzhu’ leaves.

PSY is a key regulatory enzyme that determines the total amount of carotenoid accumulation in plants. The downregulation of *PSY* leads to changes in the expression of genes involved in many metabolic pathways that encode key enzymes, such as carotenoids, gibberellin, abscisic acid and chlorophyll biosynthesis pathways, as well as the yellowing of *Oncidium* hybrid orchid leaves [46]. In this study, we isolated 2 PSY, but their expression levels are not consistent with the changes of carotenoid content, and the specific reasons need to be further studied. Then, we isolated 8 carotenoid synthesis genes (2 PDS, 2 Z-ISO, 1 ZDS, 1 CRTISO, 1 LCYE, 1 LCYB), were much higher at the T3 or T4. Chai et al. (2011) [66] found that when T-DNA in *OsCRTISO* gene encoding carotenoid isomerase was inserted, the leaves would produce “zebra’’ mutants in rice. LCYE and LCYB are key enzymes that catalyze the cyclization of lycopene, and determine the content and composition of carotenoids in plant tissues. Li et al. (2018) [46] found that the golden leaf of *ginkgo biloba* leaves is because *PPO* and *NYC/NOL* inhibit the synthesis of chlorophyll, and *Z-ISO, ZDS* and *LCYE* promote the content of carotenoids.

The above genes and α-Carotene and β-Carotene production is closely related, while CHYE, CHYB and ZEP are involved in the biosynthesis of other carotenoids [67]. We screened 13 genes related to the biosynthesis of other carotenoids, of which 7 (3 CHYB, 1 CHYE, 1 ZEP, 2 VDE) were upregulated and 5 CHYB and 1 ZEP were downregulated from T1 to T4. These genes have been verified in the flowers and fruits of many plants to participate in the regulation of carotenoid synthesis, such as carrot [68], morning glory [69], zucchini [70], strawberry [71], etc. In addition, the carotenoid content of plants is affected not only by the key enzymes of carotenoid synthesis pathway, but also by the enzymes related to the degradation pathway. There are two main carotenoid degradation pathways in plants, which are completed by the action of carotenoid dioxygenase (CCD) and NCED [72]. During the development of peony leaves, NCED gene was upregulated at leaf full green period, resulting in a decrease in carotenoid accumulation [24]. We screened 5 NCEDs, of which 1 was upregulated and 4 were downregulated from T1 to T4. In the present study, PDS, Z-ISO, ZDS, CRTISO, LCYE, LCYB, CHYE and VDE were upregulated from T1 to T4, which was consistent with the changes of carotenoid levels, suggesting that these genes are closely related to carotenoid production during the process of leaf color changes. The expression of PSY, ZEP and CHYB are irregular. These distinct gene expression patterns indicated the exceptional intricacy of the carotenoid metabolism underpinning the ‘Ziyan Gongzhu’ leaf color change.

### Other metabolic pathways affect leaf color

Chlorophyll fluorescence parameter is an important parameter reflecting the photosynthetic capacity of plants, which can reflect the photochemical reaction activity and self-protection ability of plant leaves in real time [73]. In this study, the results show that the levels of Y(II) and ETR of T1, T2 and T3 were significantly lower than T4 (Fig. 7A, B). Combining the results of the highest chlorophyll content, the largest number of chloroplasts in each cell, the largest size of chloroplasts and the most perfect development of leaves in T4 period, we guess that the abnormal development of chloroplasts also affects the photosynthesis of leaves. Conversely, NPQ level in T1 and T2 were significantly higher than T3 and T4 (Fig. 7C). A large number of experiments have proved that anthocyanins have the ability to resist excessive visible light to protect plants, especially anthocyanins located in the epidermis reduce the stress of oversaturated light on plants through light attenuation [74]. And we speculate that the high NPQ at T1 and T2 is due to the high content of anthocyanins. Based on transcriptome data, we identified 56 DEGs that are related to photosynthesis in ‘Ziyan Gongzhu’ leaves (Table S15). The DEGs are enriched in the photosynthetic pathway of plants such as (PSI, PSII), and most of these DEGs upregulated (Fig. S6). These results are consistent with our previous analysis results of chlorophyll content, chloroplast ultrastructure and chlorophyll fluorescence parameters, indicating that the leaf color of ‘Ziyan Gongzhu’ is largely affected by changes in pigment content and abnormal chloroplast development.

Sugar is the signal and precursor of anthocyanin biosynthesis [75]. One is that sugar participates in the synthesis of anthocyanins, and anthocyanins form stable anthocyanins after glycosylation; The second is that the precursor required for anthocyanin synthesis is produced by shikimic acid, and the formation of shikimic acid depends on vigorous pentose respiration, and sufficient sugar is the necessary condition for pentose respiration. In this study, we screened 25 carbohydrate DAMs, of which 16 DAMs had the lowest content in T1 (Fig. S7 and Table S16). At the same time, we also screened 211 DEGs related to carbohydrate metabolism, most of which are expressed highly in T1 and T2 (Table S16). Therefore, we speculate that these results are due to the high content of anthocyanins in T1 and T2, which have the highest carbohydrate consumption and demand. Therefore, carbohydrate metabolism in leaves may not affect the color of leaves directly, but may affect the color change of leaves by affecting the synthesis of anthocyanins.

Plant endogenous hormones control pigment synthesis by influencing regulatory genes. The promotion effect of cytokinin (CK) on anthocyanin synthesis has been verified in many plants, such as *Daucus carota*, *Rosacea* and *Brassica campestris* [76]. In this study, we screened 26 histone kinases (AHK2, AHK3 and AHK4), which are cytokinin receptors involved in anthocyanin synthesis in the cytokinin signal pathway (Table S17), of which 16 DEGs are downregulated from T1 to T4, and 10 DEGs are up-regulated, which is consistent with the results of anthocyanin content changes. Therefore, we speculate that cytokinin participates in regulating the change of leaf color of ‘Ziyan Gongzhu’ by regulating anthocyanin synthesis. The inhibition of ethylene on anthocyanin synthesis has been confirmed in many experiments. Overexpression of mutant ethylene receptor gene (*ETR1H69A*) in tobacco petals will increase anthocyanin content [77]. In this study, we screened 18 receptors DEGs that can sense ethylene signal (ETR1, ETR2, ERS1, ERS2 and EIN4, etc.), 12 of which are upregulated from T1 to T4 (Table S17), consistent with the changes in anthocyanin content, so we speculate that ethylene signal pathway may participate in the regulation of ‘Ziyan Gongzhu’ leaf color by regulating anthocyanin synthesis. The combination of ABA-responsive element (ABRE)-binding, ABF2, ABF3 and ABF4 can activate the chlorophyll decomposition in *Arabidopsis thaliana* to produce genes related to metabolism and aging, and promote ABA-mediated chlorophyll degradation [78]. In this study, we screened 7 ABFs that were up-regulated from T1 to T3 and downregulated at T4 (Table S17), which is the same result as the content of two ABAs that we also screened through non-targeted metabolomics. Therefore, we speculate that ABA-mediated chlorophyll degradation may also participate in the regulation of leaf color change of ‘Ziyan Gongzhu’. In previous studies, ethylene signal pathway inhibits carotenoid synthesis and ABA signal pathway promotes anthocyanin synthesis [79], but no related regulatory factors have been found in this study, The specific mechanism of hormones involved in regulating leaf color change of color-leafed plants needs further study.

## Conclusion

**Fig. 17:**
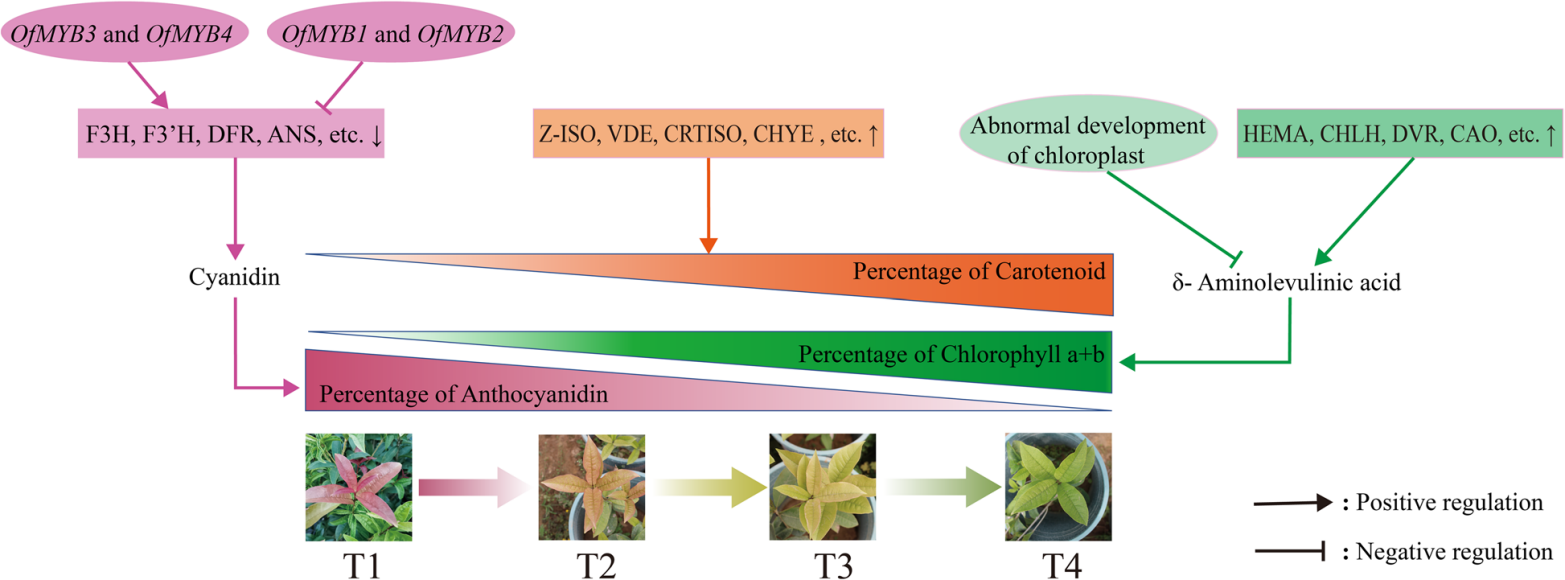
Schematic of changes in the regulatory genes and metabolites in leaf color-transition of ‘Ziyan Gongzhu’. ↑, upregulation of gene expression; ↓, downregulation of gene expression. (T1: purple red leaf (RHS 67A), T2: light purple leaf (RHS 65A), T3: yellow green leaf (RHS 1C), T4: dark green leaf (RHS N137A)

In this study, we analyzed the regulatory mechanism of four different color leaves of ‘Ziyan Gongzhu’ through physiological, transcriptome and metabolome levels. Based on our results, we provide a regulatory model for the mechanisms for leaf color changes in ‘Ziyan Gongzhu’ (Fig. 17). Overall, the content of chlorophyll a + b is low and the content of anthocyanin is high in T1 and T2 leaves, along with low expression of chlorophyll synthesis genes (HEMA, CHLG, and CAO, etc.) and high expression of anthocyanin synthesis genes (F3H, F3’H, DFR and ANS, etc.), resulting purple red and light purple in T1 and T2 leaves, respectively. It was also found that the pigment closely related to the color leaves of ‘Ziyan Gongzhu’ was cyanidin. The content of flavonoids, including anthocyanins, may be regulated by two putative MYB activators (*OfMYB3* and *OfMYB4*) and two putative MYB repressors (*OfMYB1* and *OfMYB2*). In contrast, the content of chlorophyll a + b is high and the content of anthocyanin is low in T3 and T4 leaves, along with high expression of chlorophyll synthesis genes (HEMA, CHLG, and CAO, etc.) and low expression of anthocyanin synthesis genes (F3H, F3’H, DFR and ANS, etc.), resulting yellow green and dark green in T3 and T4 leaves, respectively. And abnormal chloroplast development affects chlorophyll content in T1, T2, and T3 leaves. Although the content of carotenoids first dropped in T2 leaves, it then rapidly accumulated in T4 leaves, in sync with the increase in the expression of genes related to carotenoid biosynthesis (ZDS, LHYB, and ZEP, for example). Meanwhile, the chlorophyll fluorescence parameter NPQ is higher in T1 and T2, ETR, Y(II) is the highest in T4, and the upregulation of most photosynthetic related DEGs once again confirmed that leaf color is largely affected by changes in pigment content and abnormal chloroplast development. Analysis of carbohydrate and hormone-related DAMs and DEGs found that they may participate in the regulation of leaf color change of ‘Ziyan Gongzhu’ by affecting pigment synthesis. Our results pave the way for a comprehensive knowledge of the regulatory processes governing leaf color in ‘Ziyan Gongzhu’ and identify possible genes for application in the molecular breeding of colored-leaf cultivars.

### Supplementary Information

**Additional file 1:  Table S1.** 26 MYBs from other plant species used for phylogenetic analysis.

**Additional file 2:  Table S2.** Primers used for RT-PCR analysis in this study.

**Additional file 3:  Table S3.** Summary of the RNA-Seq data collected from the leaves of “Ziyan Gongzhu”. 

**Additional file 4:  Table S4.** Summary of clean reads and genes mapped to the reference genome from “Ziyan Gongzhu”.

**Additional file 5:  Table S5.** List of known genes identified in “Ziyan Gongzhu” leaves.

**Additional file 6:  Table S6.** List of novel genes identified in “Ziyan Gongzhu” leaves.

**Additional file 7:  Table S7.** List of enriched KEGG for DEGs in “Ziyan Gongzhu”.

**Additional file 8:  Table S8.** Four differentially expressed OfMYBs.

**Additional file 9:  Table S9.** Metabolites identified in the four “Ziyan Gongzhu” leaves samples in NEG and POS. 

**Additional file 10:  Table S10.** List of enriched KEGG for DAMs in “Ziyan Gongzhu”.

**Additional file 11:  Table S11.** List of common KEGG pathways for DAMs and DEGs in “Ziyan Gongzhu”.

**Additional file 12:  Table S12.** List of DEGs and DAMs involved in flavonoid biosynthesis in “Ziyan Gongzhu”.

**Additional file 13:  Table S13.** List of DEGs and DAMs involved in chlorophyll and heme metabolism in “Ziyan Gongzhu”.

**Additional file 14:  Table S14.** List of DEGs and DAMs involved in carotenoid metabolism in “Ziyan Gongzhu”.

**Additional file 15:  Table S15.** List of DEGs related to photosynthesis in “Ziyan Gongzhu”.

**Additional file 16:  Table S16.** List of DEGs and DAMs related to carbohydrate metabolism in “Ziyan Gongzhu”.

**Additional file 17:  Table S17.** List of DEGs related to hormone signal transduction in “Ziyan Gongzhu”.

**Additional file 18:** **Fig. S1.** GO pathway enrichment analysis of DEGs between T1 vs T2 (A), T1 vs T3 (B), T1 vs T4 (C), T2 vs T3 (D), T2 vs T4 (E) and T3 vs T4 (F) of “Ziyan Gongzhu”. (T1: purple red leaf (RHS 67A), T: stage, T2: light purple leaf (RHS 65A), T3: yellow green leaf (RHS 1C), T4: dark green leaf (RHS N137A). **Fig. S2.** Principal component analysis of the four “Ziyan Gongzhu” leaves samples in NEG (A) and POS (B). (T1: purple red leaf (RHS 67A), T2: light purple leaf (RHS 65A), T3: yellow green leaf (RHS 1C), T4: dark green leaf (RHS N137A). **Fig. S3.** Hierarchical heatmap clustering analysis of the four “Ziyan Gongzhu” leaves samples using the metabolite concentration data in NEG (A) and POS (B). (T1: purple red leaf (RHS 67A), T2: light purple leaf (RHS 65A), T3: yellow green leaf (RHS 1C), T4: dark green leaf (RHS N137A). **Fig. S4.** Correlation analysis of the four “Ziyan Gongzhu” leaves samples in NEG (A) and POS (B). (T1: purple red leaf (RHS 67A), T2: light purple leaf (RHS 65A), T3: yellow green leaf (RHS 1C), T4: dark green leaf (RHS N137A). **Fig. S5.** KEGG pathway enrichment analysis of DAMs between T1 vs T2 (A), T1 vs T3 (B), T1 vs T4 (C), T2 vs T3 (D), T2 vs T4 (E) and T3 vs T4 (F) of “Ziyan Gongzhu”. (T1: purple red leaf (RHS 67A), T: stage, T2: light purple leaf (RHS 65A), T3: yellow green leaf (RHS 1C), T4: dark green leaf (RHS N137A). **Fig. S6.** Heat map of differentially expressed genes (DEGs) related to photosynthesis in leaves of “Ziyan Gongzhu” at the four developmental stages according to hierarchical cluster analysis. Blue indicates the lowest expression; white indicates intermediate expression and red indicates the highest expression. A colour scale bar is shown at the top-right comer of the figure and corresponds to the values of the mean-centred log2-transformed fragments per kilobase per million reads (FPKM). (T1: purple red leaf (RHS 67A), T: stage, T2: light purple leaf (RHS 65A), T3: yellow green leaf (RHS 1C), T4: dark green leaf (RHS N137A). **Fig. S7.** Heat map of differential abundant metabolites (DAMs) related to carbohydrate in leaves of “Ziyan Gongzhu” at the four developmental stages according to hierarchical cluster analysis. Blue indicates the lowest abundant; white indicates intermediate abundant and red indicates the highest abundant. (T1: purple red leaf (RHS 67A), T: stage, T2: light purple leaf (RHS 65A), T3: yellow green leaf (RHS 1C), T4: dark green leaf (RHS N137A).

## Data Availability

All data generated or analyzed during this study are included in this published article, its supplementary information files and publicly available repositories. The raw RNA-Seq reads are available at the NCBI Sequence Read Archive (SRA): PRJNA942013. The DEGs used in phylogenetic analyses are available at GenBank: OQ594390 (*OfMYB1*), OQ594391 (*OfMYB2*), OQ594392 (*OfMYB3*), OQ594393 (*OfMYB4*).

## Abbreviations

PAL: Phenylalanine ammonia-lyase
UGT: Glucose-flavonoid 3-o-glcosyl-transferase
C4H: Cinnamic acid hydroxylase
4CL: Coumadin CoA ligase
CHS: Chalcone synthase
CHI: Chalcone isomerase
F3H: Flavonoid 3-hydroxylase
DEG: Differentially expressed gene
FDR: False discovery rate
MYB: V-myb avian myeloblastosis viral oncogene homolog
GluTR: Glutamyl-trna reductase
CPOX: Coproporphyrinogen III oxidase
MgCh: Magnesium chelatase
DVR: 3,8-Divinyl protochlorophyllide a 8-vinyl reductase
POR: Protochlorophyllide oxidoreductase
PSY: Phytoene synthase
ZDS: ζ-Carotene desaturase
Z-ISO: ζ-Carotene isomerase
LCYE: Lycopene ε-cyclase
BCH: β-Carotene hydrolase
CYP97A: Cytochrome P450 carotene β-hydroxylase
F3’H: Flavonoid 3’-hydroxylase
F3′5’H: Flavonoid 3′ 5’-hydroxylase
DRF: Dihydroflavonol 4-reductase
ANS: Anthocyanin synthase
GO: Gene ontology
KEGG: Kyoto encyclopedia of genes and genomes
TF: Transcription factor
DAM: Differentially abundant metabolite
RT-PCR: Real-time reverse transcription-pcr
Chl: Chlorophyll
ALA: 5-Aminolevulinic acid
PPOX: Protoporphyrinogen oxidase
MgP IX: Mg-protoporphyrin IX
CAO: Chlorophyllide a oxygenase
CHLG: Chlorophyll synthase
PDS: Phytoene desaturase
GGPP: Geranylgeranyl diphosphate
CRTISO: Carotene isomerase
LCYB: Lycopene β-cyclase
ZEP: Zeaxanthin epoxidase
CYP97C: Cytochrome P450 carotene ε-hydroxylase
