# Supplementary material for: Mechanisms for leaf color changes in Osmanthus fragrans ‘Ziyan Gongzhu’ using physiology, transcriptomics and metabolomics
Source: BMC Plant Biol. 2023 Sep 27;23:453. doi: 10.1186/s12870-023-04457-8 (PMC10523669; doi:10.1186/s12870-023-04457-8)
Supplement: Supplementary file 18 — Additional file 18: Fig. S1. GO pathway enrichment analysis of DEGs between T1 vs T2 (A), T1 vs T3 (B), T1 vs T4 (C), T2 vs T3 (D), T2 vs T4 (E) and T3 vs T4 (F) of “Ziyan Gongzhu”. (T1: purple red leaf (RHS 67A), T: stage, T2: light purple leaf (RHS 65A), T3: yellow green leaf (RHS 1C), T4: dark green leaf (RHS N137A). Fig. S2. Principal component analysis of the four “Ziyan Gongzhu” leaves samples in NEG (A) and POS (B). (T1: purple red leaf (RHS 67A), T2: light purple leaf (RHS 65A), T3: yellow green leaf (RHS 1C), T4: dark green leaf (RHS N137A). Fig. S3. Hierarchical heatmap clustering analysis of the four “Ziyan Gongzhu” leaves samples using the metabolite concentration data in NEG (A) and POS (B). (T1: purple red leaf (RHS 67A), T2: light purple leaf (RHS 65A), T3: yellow green leaf (RHS 1C), T4: dark green leaf (RHS N137A). Fig. S4. Correlation analysis of the four “Ziyan Gongzhu” leaves samples in NEG (A) and POS (B). (T1: purple red leaf (RHS 67A), T2: light purple leaf (RHS 65A), T3: yellow green leaf (RHS 1C), T4: dark green leaf (RHS N137A). Fig. S5. KEGG pathway enrichment analysis of DAMs between T1 vs T2 (A), T1 vs T3 (B), T1 vs T4 (C), T2 vs T3 (D), T2 vs T4 (E) and T3 vs T4 (F) of “Ziyan Gongzhu”. (T1: purple red leaf (RHS 67A), T: stage, T2: light purple leaf (RHS 65A), T3: yellow green leaf (RHS 1C), T4: dark green leaf (RHS N137A). Fig. S6. Heat map of differentially expressed genes (DEGs) related to photosynthesis in leaves of “Ziyan Gongzhu” at the four developmental stages according to hierarchical cluster analysis. Blue indicates the lowest expression; white indicates intermediate expression and red indicates the highest expression. A colour scale bar is shown at the top-right comer of the figure and corresponds to the values of the mean-centred log2-transformed fragments per kilobase per million reads (FPKM). (T1: purple red leaf (RHS 67A), T: stage, T2: light purple leaf (RHS 65A), T3: yellow green leaf (RHS 1C), T4: dark green leaf (RHS N [file 12870_2023_4457_MOESM18_ESM.pdf]

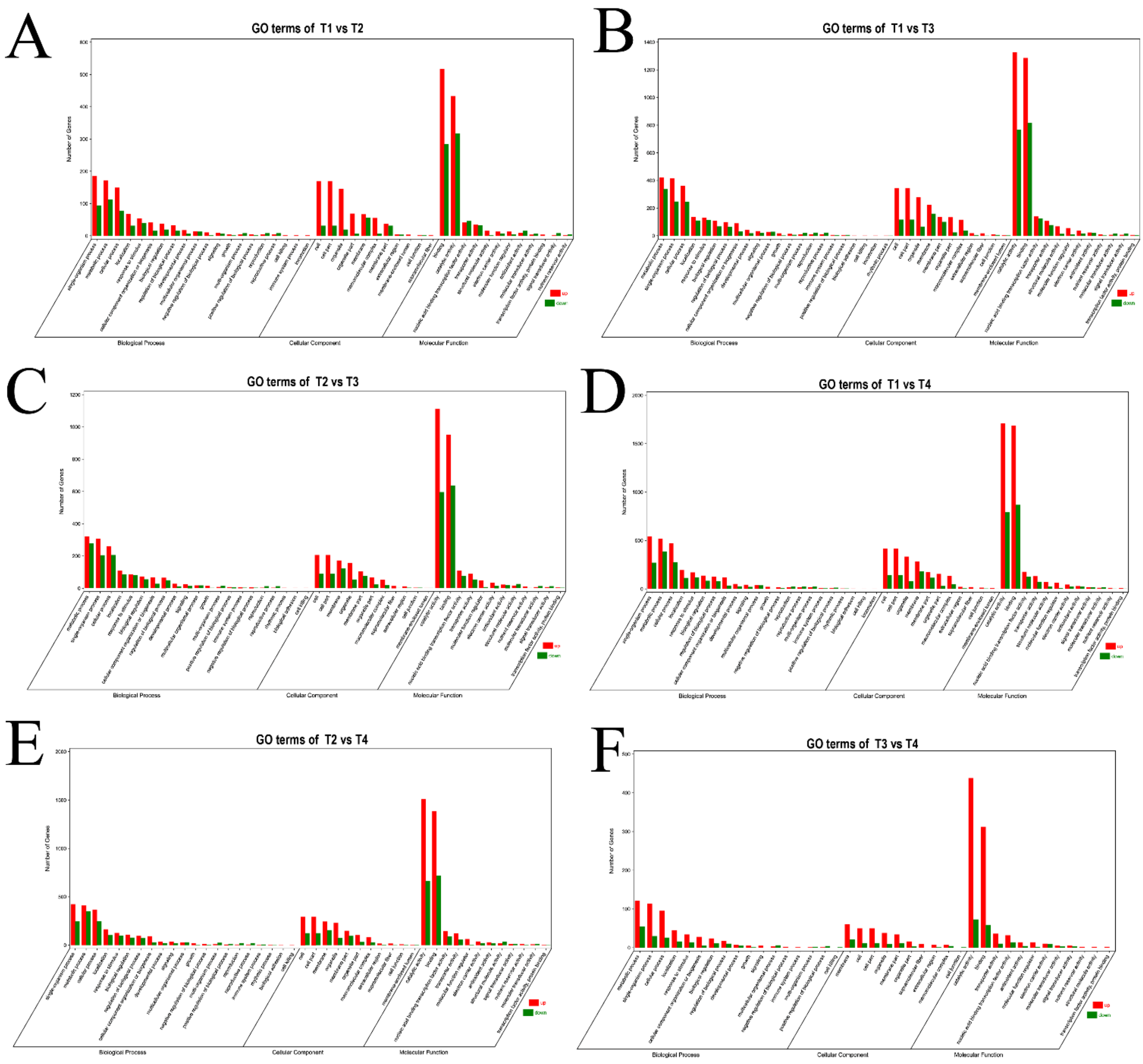

Fig. S1 GO pathway enrichment analysis of DEGs between T1 vs T2 (A), T1 vs T3 (B), T1 vs T4 (C), T2 vs T3 (D), T2 vs T4 (E) and T3 vs T4 (F) of “Ziyan Gongzhu”. (T1: purple red leaf (RHS 67A), T: stage, T2: light purple leaf (RHS 65A), T3: yellow green leaf (RHS 1C), T4: dark green leaf (RHS N137A).

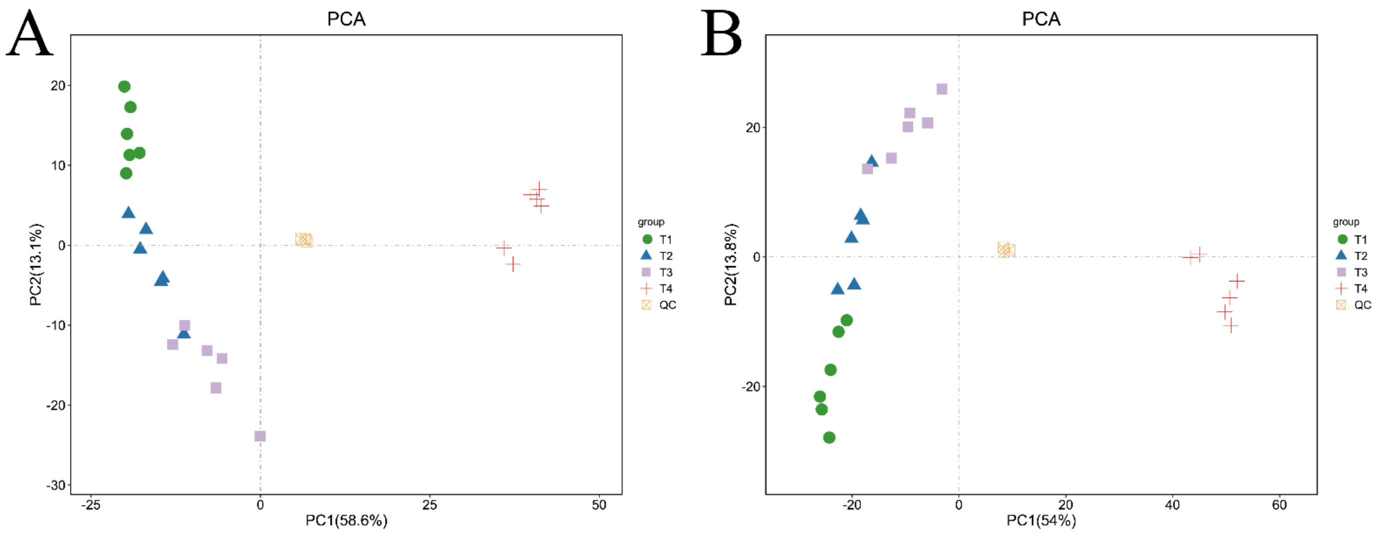

Fig. S2 Principal component analysis of the four “Ziyan Gongzhu” leaves samples in NEG (A) and POS (B). (T1: purple red leaf (RHS 67A), T2: light purple leaf (RHS 65A), T3: yellow green leaf (RHS 1C), T4: dark green leaf (RHS N137A).

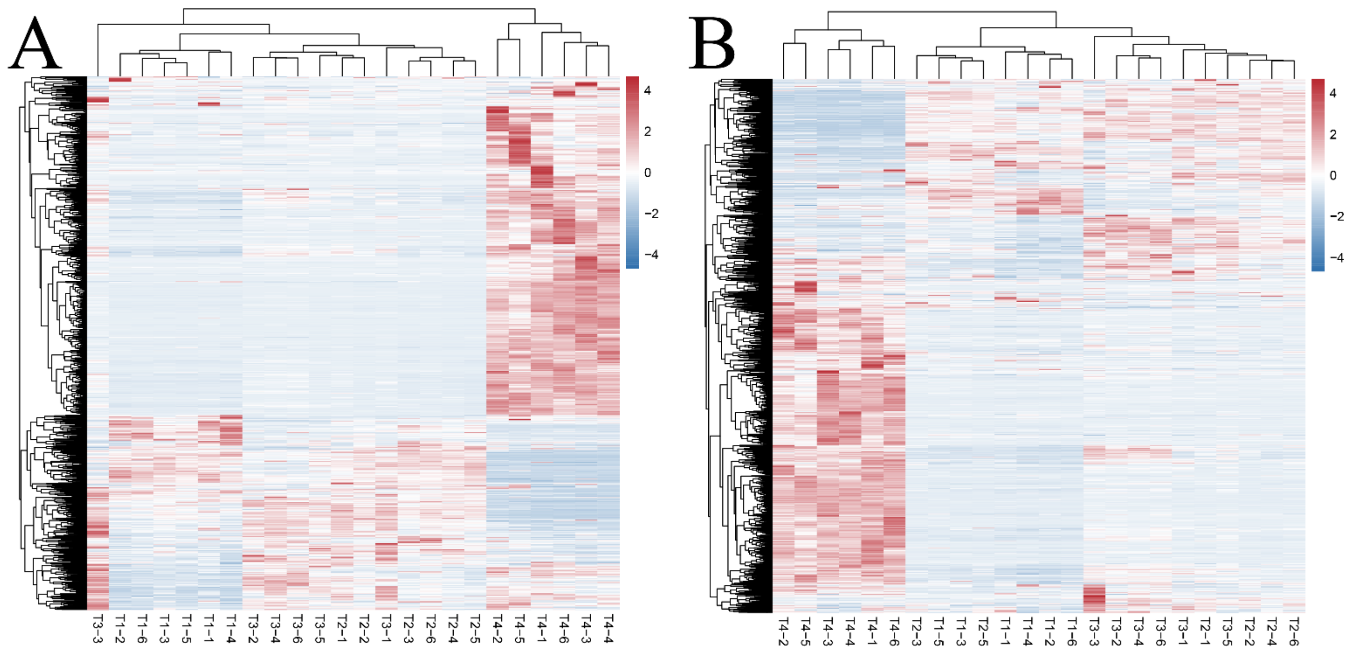

Fig. S3 Hierarchical heatmap clustering analysis of the four “Ziyan Gongzhu” leaves samples using the metabolite concentration data in NEG (A) and POS (B). (T1: purple red leaf (RHS 67A), T2: light purple leaf (RHS 65A), T3: yellow green leaf (RHS 1C), T4: dark green leaf (RHS N137A).

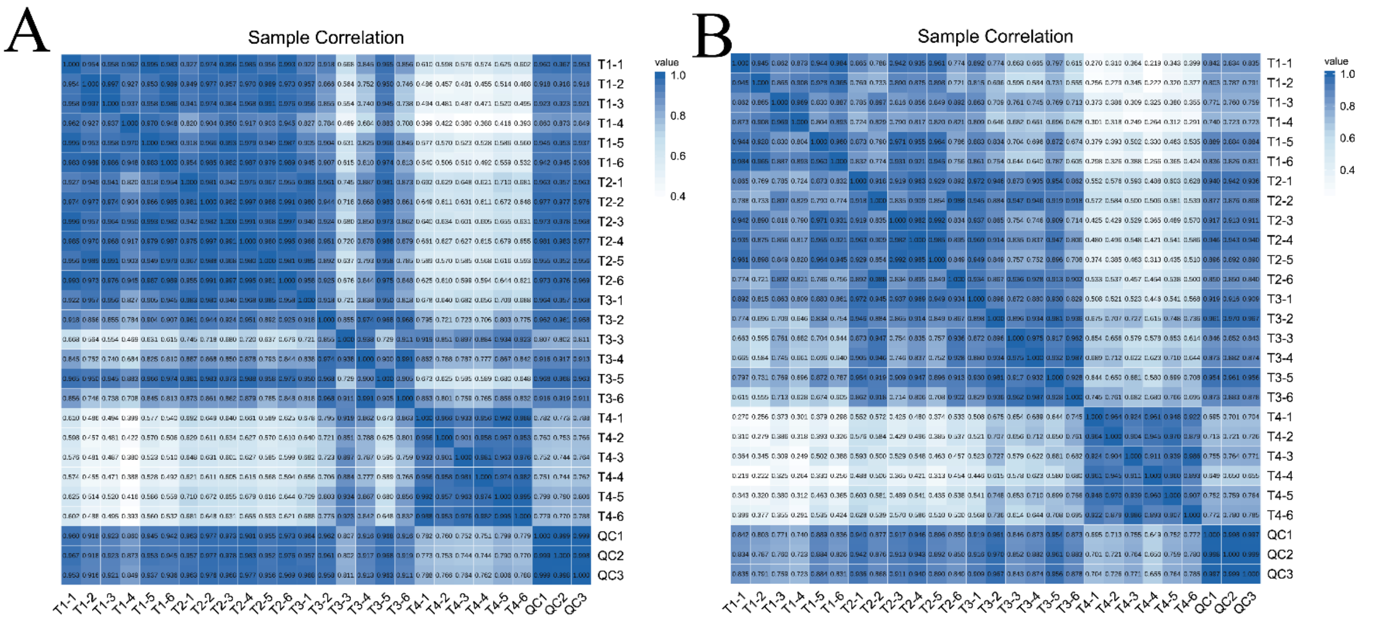

Fig. S4 Correlation analysis of the four "Ziyan Gongzhu" leaves samples in NEG (A) and POS (B). (T1: purple red leaf (RHS 67A), T2: light purple leaf (RHS 65A), T3: yellow green leaf (RHS 1C), T4: dark green leaf (RHS N137A).

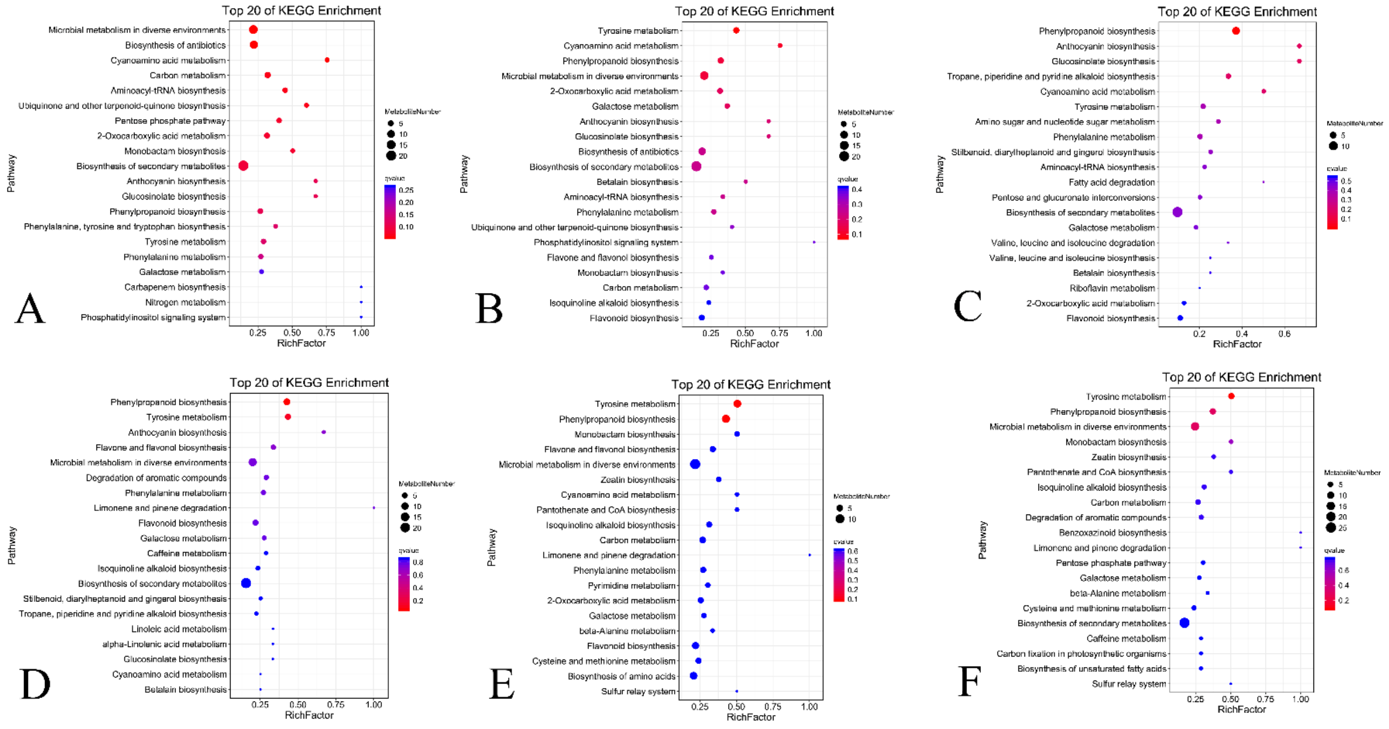

Fig. S5 KEGG pathway enrichment analysis of DAMs between T1 vs T2 (A), T1 vs T3 (B), T1 vs T4 (C), T2 vs T3 (D), T2 vs T4 (E) and T3 vs T4 (F) of "Ziyan Gongzhu". (T1: purple red leaf (RHS 67A), T: stage, T2: light purple leaf (RHS 65A), T3: yellow green leaf (RHS 1C), T4: dark green leaf (RHS N137A).

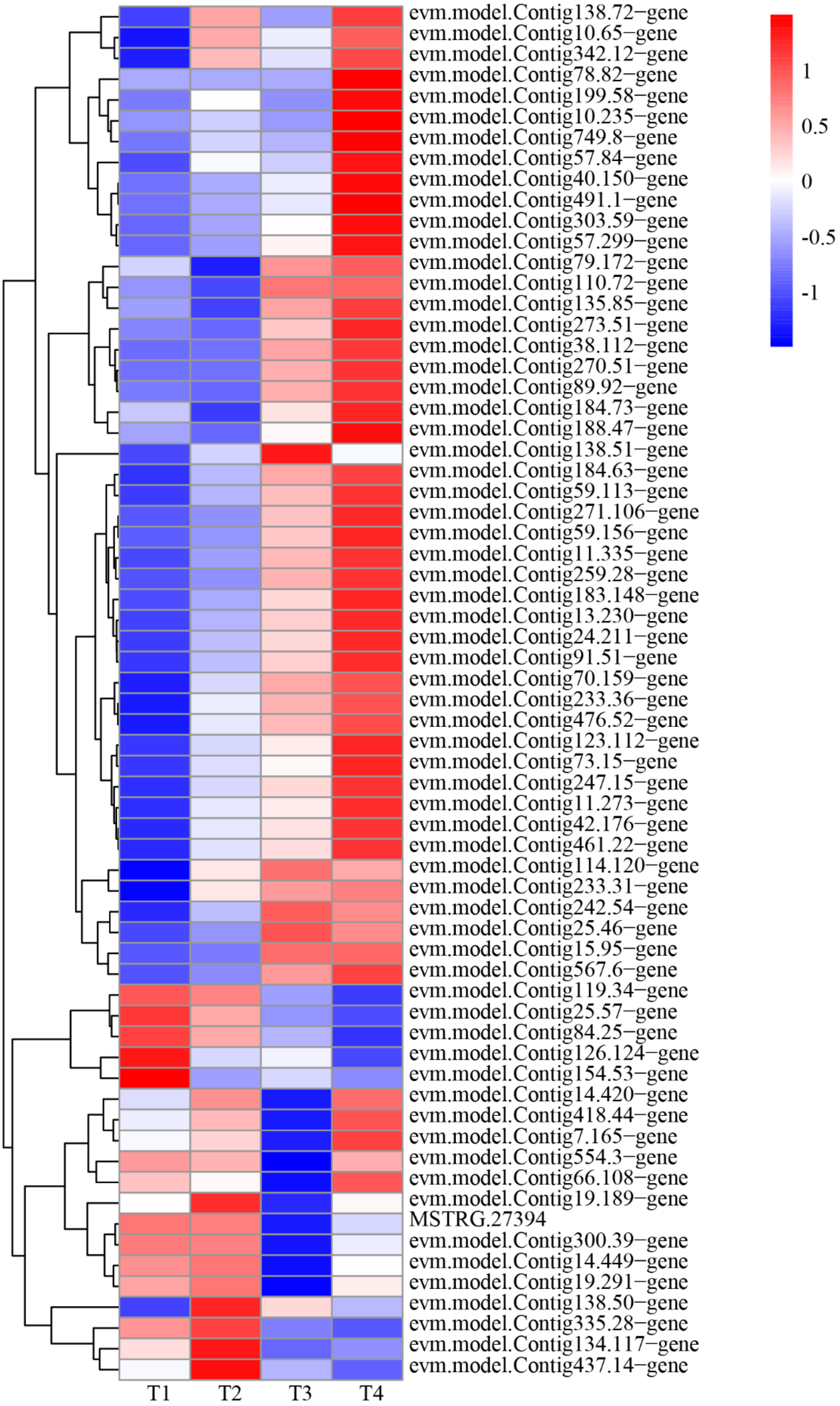

Fig. S6 Heat map of differentially expressed genes (DEGs) related to photosynthesis in leaves of "Ziyan Gongzhu" at the four developmental stages according to hierarchical cluster analysis. Blue indicates the lowest expression; white indicates intermediate expression and red indicates the highest expression. A colour scale bar is shown at the top-right comer of the figure and corresponds to the values of the mean-centred log_2-transformed fragments per kilobase per million reads (FPKM). (T1: purple red leaf (RHS 67A), T: stage, T2: light purple leaf (RHS 65A), T3: yellow green leaf (RHS 1C), T4: dark green leaf (RHS N137A).

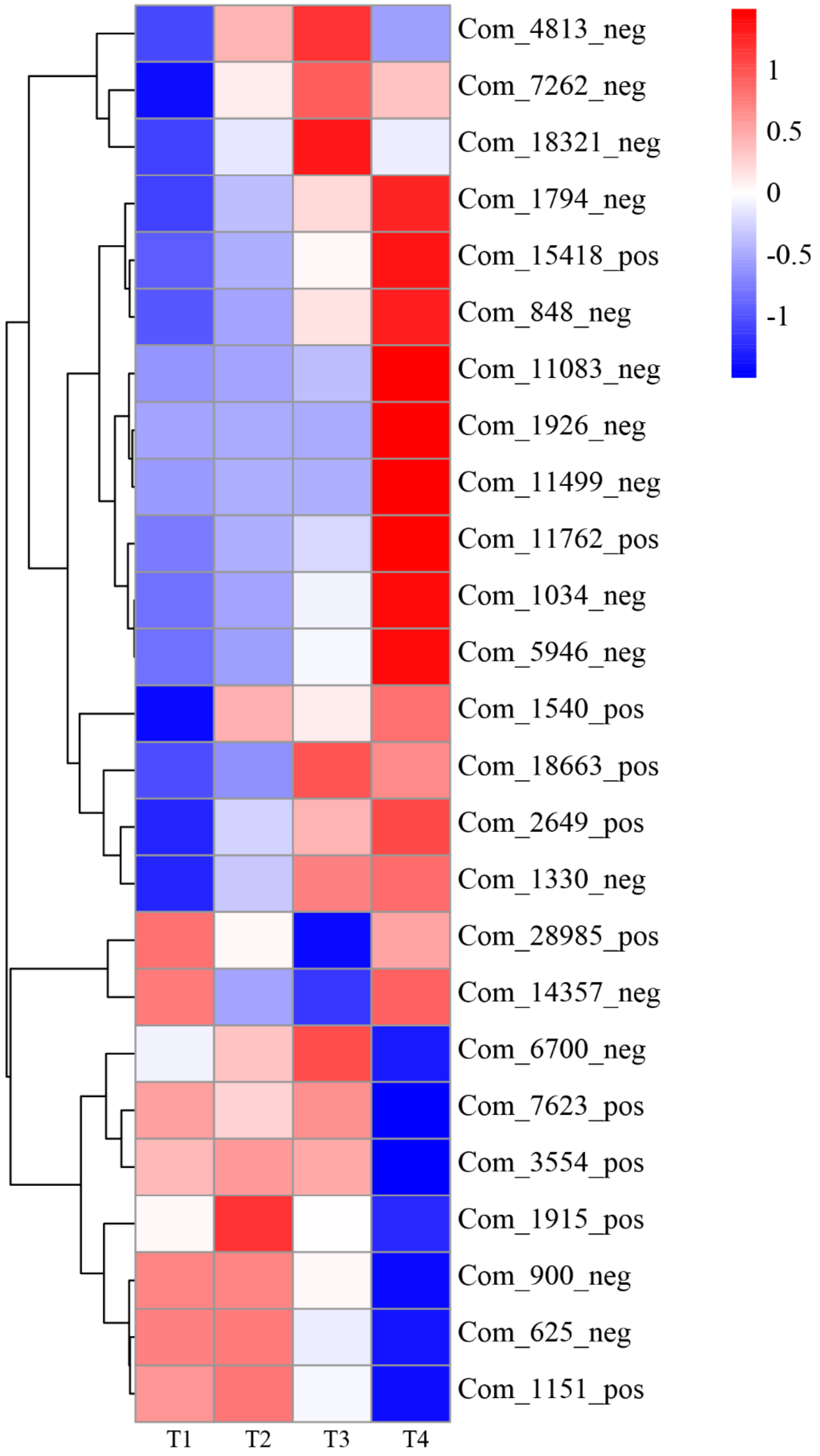

Fig. S7 Heat map of differential abundant metabolites (DAMs) related to carbohydrate in leaves of "Ziyan Gongzhu" at the four developmental stages according to hierarchical cluster analysis. Blue indicates the lowest abundant; white indicates intermediate abundant and red indicates the highest abundant. (T1: purple red leaf (RHS 67A), T: stage, T2: light purple leaf (RHS 65A), T3: yellow green leaf (RHS 1C), T4: dark green leaf (RHS N137A).
